# Genome-wide association analysis reveals genetic variations and candidate genes associated with tannin and starch contents in sorghum

**DOI:** 10.1186/s12864-026-12914-5

**Published:** 2026-05-04

**Authors:** Xinqi Fan, Du Liang, Chenchen Wang, Yizhong Zhang, Qi Guo, Huiyan Wang, Chunhong Li, Xinlian Shen, Qingshan Liu, Peng Xu

**Affiliations:** 1 Shanxi Houji Laboratory, Sorghum Research Institute, Shanxi Agricultural University, Yuci, 030600 China; 2 College of Agriculture, Shanxi Agricultural University, Taigu, 030801 China; 3 Department of Social Services, Shanxi Agricultural University, Taigu, 030801 China; 4 Key Laboratory of Cotton and Rapeseed (Nanjing), Ministry of Agriculture, Nanjing, 210014 China; 5 The Institute of Industrial Crops, Jiangsu Academy of Agricultural Sciences, Nanjing, 210014 China

**Keywords:** Sorghum, Genome-wide association analysis, Tannin content, Starch content, Chalcone synthase, Glucose-6-phosphate translocator

## Abstract

**Background:**

Sorghum is the key grain for brewing distilled liquors. Grain composition, particularly tannin and starch levels, is closely linked to the sensory characteristics and yield of the final product. Developing elite sorghum cultivars with suitable tannin and elevated starch content is thus essential for producing liquors with diverse and distinct aroma profiles. Investigating the natural variations of grain composition in sorghum germplasms and identifying underlying genetic loci can facilitate the improvement of sorghum’s nutritional value.

**Results:**

This study established near-infrared reflectance spectrometer (NIRS) non-destructive calibration models for determining sorghum tannin and starch contents based on phenotypic identification of 214 representative sorghum cultivars. A genome-wide association study (GWAS) of tannin and starch contents was performed based on two years and two locations of phenotypic data. A total of 18 associations with 338 single nucleotide polymorphisms (SNPs) scattered over 4 chromosomes were detected significantly associated with tannin and starch contents. Among these, two associations for tannin content and two associations for starch content were expressed in different environments, indicating that they were likely to be stable quantitative trait loci (QTLs). Through linkage disequilibrium (LD) block and haplotype analysis, a promising gene, Sobic.003G118266, which encodes chalcone synthase (CHS), was identified as orthologous to structural genes in known tannin synthesis pathways; while another promising gene, Sobic.004G353100, annotated as a glucose-6-phosphate translocator (G6PT), was determined to be a putative regulatory node in plant starch synthesis.

**Conclusion:**

These results provided important genetic variations and candidate genes to accelerate the improvement of sorghum breeding for liquor-brewing quality related traits.

**Supplementary Information:**

The online version contains supplementary material available at 10.1186/s12864-026-12914-5.

## Background

Sorghum (*Sorghum bicolor* L.), with a cultivation history spanning thousands of years in China, stands as one of the earliest domesticated cereal crops in the country. It long served as a staple food for people in northern China. As food security improved and living standards rose, sorghum transitioned from a dietary staple to an industrial raw material, now extensively utilized in liquor production, animal feed, vinegar manufacturing, and other applications, continuing to play a vital role in the national economy and agricultural production. Particularly significant is its use in liquor production, which sustains relatively stable consumption volumes of sorghum in China [1]. Sorghum is primarily cultivated in the Southwest, North, and Northeast regions, with over 80% of sorghum grains annually used as raw material for liquor production [2]. Renowned Chinese liquors representing the three basic aroma types including Moutai, Wuliangye, and Fenjiu rely on sorghum as their primary fermentation substrate, highlighting the critical importance of grain quality traits for liquor production .

The nutritional components in sorghum grains, such as tannin and starch contents, are closely related to the aroma type, taste, and alcohol yield of the liquor. Tannins, also known as proanthocyanidins, are primarily found in the outer seed coat of sorghum. During baijiu fermentation, they inhibit the growth of harmful microorganisms while their derivatives (such as syringic acid and syringaldehyde) enhance the aromatic flavor of the liquor [3]. Starch serves as the primary saccharification substrate in brewing, with its content showing a significantly positive correlation with alcohol production rate. As starch quality directly impacts the economic efficiency of the liquor industry, it has become a crucial quality indicator monitored by distilleries. The brewing quality and ultimate utilization value of sorghum grains are predominantly determined by tannin and starch characteristics, which exhibit substantial compositional variations. Only premium-quality sorghum can yield high-grade liquor. Therefore, breeding elite sorghum varieties with optimized tannin levels and high starch content is essential to meet the diverse requirements of the liquor-brewing industry .

Sorghum quality is crucial for both consumers and processing enterprises. Research into the molecular basis can elucidate the molecular mechanisms underlying sorghum quality formation. Studying the molecular basis of traits such as tannin and starch contents provides a scientific foundation for breeding high-quality sorghum varieties. Simultaneously, molecular marker-assisted selection and genetic improvement can accelerate the process of sorghum quality enhancement, thereby improving its economic and nutritional values. Over the last two decades, around 150 studies identifying almost 6000 QTLs for over 220 traits have been published in sorghum [4]. Despite extensive QTL mapping efforts in sorghum, most identified loci relate to agronomic and stress-resilience traits, whereas grain quality traits relevant to liquor brewing remain comparatively underexplored, largely due to the technical challenges associated with accurate phenotypic assessment [5–7]. Existing research has accumulated substantial genetic information on grain brewing quality, but this knowledge has not been fully utilized in sorghum improvement programs. With the increase in sequencing studies, researchers will have access not only to high-resolution sequence data genome-wide but also to estimated haplotype information. More work needs to be done to precisely identify genes responsible for natural variation of grain composition.

Sorghum grains are rich in condensed tannins, which influence seed dormancy [8], protect against grain mold [9], and deter predation by birds and insects [10, 11]. Tannins belong to flavonoids which are secondary metabolites in higher plants known for the pigmentation in flowers, fruits, and seeds [12], involving in dozens of structural genes and multiple regulatory genes. To date, only a limited number of regulatory genes controlling tannin accumulation have been functionally characterized in sorghum [10, 13–16]. As the primary component of grains, starch is the most critical component in cereal grains, providing energy for humans and livestock and accounting for approximately 70% of the dry grain weight [17]. Cereal starch consists of two types: amylose and amylopectin. The ratio of these two components plays a decisive role in determining grain structure and quality. The biosynthesis and assembly of starch in cereals are catalyzed by various key enzymes, yet in sorghum, only the functional enzyme granule-bound starch synthase involved in starch synthesis has been characterized [18]. Consequently, the genetic regulatory mechanisms underlying liquor-brewing quality related traits remain poorly understood, and the genetic potential of these traits has yet to be fully exploited in sorghum breeding.

Investigating the natural variation of grain composition in sorghum germplasms and identifying underlying genetic loci can facilitate the improvement of sorghum’s nutritional value. Genome-wide association studies (GWAS) have been proven to be a powerful tool for deciphering the genetic basis of complex traits in plants. This approach can evaluate multiple alleles at individual loci within natural populations, delivering higher mapping resolution than linkage mapping, which is limited to assessing restricted loci in biparental populations and captures narrower levels of allelic diversity. In sorghum, GWAS has successfully identified quantitative trait loci (QTLs) for various traits [19–25]. By integrating high-density single nucleotide polymorphisms (SNPs) data with precise phenotyping, this study provides new insights into the allelic architecture of tannin and starch accumulation in sorghum grains, offering valuable targets for molecular breeding of liquor-specific sorghum varieties.

## Materials and methods

### Plant materials

A collection of 214 sorghum cultivars were selected to assemble a GWAS panel in this study. These cultivars were collected from Sorghum Germplasm Resource Conservation Bank of Sorghum Research Institute of Shanxi Agricultural University and Jiangsu Academy of Agricultural Sciences. Detailed information of the 214 germplasm resources was provided in Supplementary Table S1. The 214 materials were categorized by geographic location as follows: China, India, the United States, Japan, Russia, Africa and Mexico. In 2023, the 214 accessions were planted at the Lishui Plant Experiment Station, Nanjing, China. In 2024, the GWAS panel were grown at the Lishui Plant Experiment Station, Nanjing, China and the Dongbai Plant Experiment Station, Yuci, China. All experiments used a randomized complete block design with two replications. To avoid cross-pollination, all plants were bagged before flowering. The main stem panicles from each plot were harvested after heading and sun-dried. Seeds were threshed and stored in dry containers at room temperature.

### Acquisition of near-infrared spectroscopic data and chemical values

The 214 sorghum germplasm planted at the Lishui Plant Experiment in 2023 were utilized to establish the near-infrared reflectance spectrometer (NIRS) models. Near-infrared reflectance spectra of intact sorghum grains were collected using a near-infrared reflectance spectrometer (FOSS DA1650) across the spectral range of 930–1650 nm. The scanning was performed at intervals of 2 nm, with 32 scans per measurement. The reflectance intensity of the samples was recorded and converted into absorbance (R). Each sample was scanned three times, and the average value was calculated and transformed into log(1/R) for storage in a computer.

Tannin content was quantified through using Chinese national standard GB/T 15,686–2008. 200 mg of milled sorghum grain flour was weighed and dissolved in 10 mL of 75% dimethylformamide (DMF) solution for 1 h at room temperature, with vortex mixing at 5-minute intervals. After centrifugation, the supernatant was collected and stored protected from light. The supernatant was aliquoted into two test tubes (Tube 1 and Tube 2): Tube 1: Distilled water and ammonia solution were added, followed by vortex mixing and incubation at 25–30 °C for 10 min. The absorbance value A1 of the sample solution was measured at 525 nm using a spectrophotometer. Tube 2: Distilled water, ferric ammonium citrate solution, and ammonia solution were added, vortex-mixed, and incubated at 25–30 °C for 10 min. Using water as a blank, the absorbance value A2 of the sample solution was measured at 525 nm. The tannin content was calculated using a tannic acid calibration curve established on a dry weight basis.

Starch content was estimated by industrial standard of Ministry of Agriculture and Rural Affairs NY/T 11-1985. 30 mg of milled sorghum sample was weighed into centrifuge tubes, followed by the addition of 0.7 mL of 80% ethanol solution and thorough mixing. The mixture was incubated in a water bath at 70 °C for 2 h. After centrifugation at 12,000 ×g for 10 min, the supernatant was discarded, and the pellet was resuspended in 80% ethanol and vortex-mixed vigorously. Subsequently, 1 mL of thermostable α-amylase was added, and the mixture was treated in a boiling water bath for 10 min. After cooling, glucosidase was added, and the sample was incubated at 50 °C for 30 min. Following centrifugation at 3,000 × g for 10 min, the supernatant was transferred to a new centrifuge tube. A glucose oxidase-peroxidase-4-aminoantipyrine (GOPOD) buffer mixture was added to the supernatant and incubated at 50 °C for 30 min. The optical density (OD) was measured at 510 nm using a spectrophotometer.

### Establishment of calibration models for nondestructive determination of tannin and starch contents

FOSS WinISI III software was used to analyze spectral data. Abnormal values in the spectral data were removed by calculating the Global H (where Global H ≥ 2). Finally, a calibration sample set consisting of 126 samples, which are both similar and capable of representing the maximum differences among spectra, was obtained for modeling. The spectral data were preprocessed using various combinations of mathematical treatments (derivative, gap, smooth, second-order smooth) and scatter correction techniques (Standard normal variate (SNV) combined with detrending), followed by calibration using the Modified Partial Least Squares (MPLS) method. The optimal calibration model was selected by evaluating the correlation coefficient (RSQ) between the near-infrared predicted data and chemical standard data for different calibration models, the mean standard error of cross validation (SECV) between the NIR predicted values and the chemically determined values for varieties not included in the calibration set, and the mean cross-validation correlation coefficient (1-VR) between the NIR predicted values and the chemically determined values for varieties not included in the calibration set. The NIRS models were then validated using 30 samples outside the calibration sample set. The predictive performance of the calibration model was evaluated based on the standard error of prediction (SEP) and the residual prediction deviation (RPD).

### Phenotype data analysis

The successfully established NIRS models were used to determine the tannin and starch contents of sorghum germplasm in each environment. The average trait values in each environment were used for phenotypic analysis and GWAS analysis. The best linear unbiased predictions (BLUPs) values were calculated using the *lme4* and *nlme* packages in R. Genotype was treated as a random effect, while environment and replication were treated as fixed effects. Statistical analysis of the phenotype of the tannin and starch contents was performed by SPSS. *H*^*2*^ of each trait was calculated from variance components. The REML method of PROC VARCOMP in SAS/STAT software (SAS Institute Inc., Cary, NC, USA) was conducted to estimate the variance components.

### SNP genotyping, population structure, relative kinship, principal component analysis (PCA), and linkage disequilibrium (LD) and GWAS

The population was genotyped using the resequencing method. All DNA samples were extracted using the DNAsecure Plant Kit (Qiagen, Cat. No. DP320). Libraries were constructed with the Truseq Library Preparation Kit (FC-121-4001). DNA fragments were first subjected to end repair, A-tailing, and adapter ligation, followed by purification and PCR amplification. Library quality was assessed using a Bioanalyzer (Agilent, Santa Clara, USA), and PCR products were quantified with a Qubit 3.0 Fluorometer (Invitrogen, Carlsbad, USA). Final libraries were sequenced on the Illumina HiSeq X Ten platform to generate 150 bp paired-end reads. Raw sequencing data were filtered using fastp-0.20.1 software to remove adapter-containing reads, low-quality reads, and reads with unrecognized bases. Sequence alignment and variant calling were performed using BWA [26] and GATK software [27], with the reference genome sourced from the NCBI database (BTx623 v3.1). Variant sites were filtered using Plink software under the criteria of minor allele frequency (MAF) ≥ 0.05, SNPs with > 5% missing genotypes and samples with > 10% missing genotypes.

Population structure (Q) was determined using Admixture software [28] with filtered SNP markers. The number of subpopulations was determined by the cross-validation error. Relative pairwise kinship (K) was calculated by SPAGeDi [29]. PCA was analyzed using the TASSEL 5.0 [30]. The LD was also calculated with Plink [31] using the SNP data of whole genomes and each chromosome. The TASSEL 5.0 software package was employed to construct association tests of tannin and starch contents. The MLMs was performed by simultaneously accounting for multiple levels of Q-matrix and K-matrix according to the methods [32]. We defined the whole-genome significant cutoff with the adjusted Bonferroni test threshold, which was set as P = − log10 (1/545,350) = 5.74.The LD block was defined with LDBlockShow software [33]. Specifically, a QTL was considered stable if it was detected in at least two individual environments, or in at least one environment and in the BLUP analysis across environments.

## Results

### Establishment of calibration models for determination of tannin and starch contents

The tannin and starch contents in the calibration set samples covered a wide range, providing a solid foundation for establishing the NIRS models. The spectral data and chemical values of the calibration set samples were correlated to determine the qualitative or quantitative relationship between them. For tannin, the coefficient of determination in calibration (RSQ) was 0.916, with a standard error of calibration (SEC) 0.191. The coefficient of determination in cross validation (1-VR) was 0.894, and the standard error of cross validation (SECV) was 0.201. External validation of the calibration model was performed using the tannin validation set samples. The coefficient of determination (R^2^) between the predicted values and the chemically measured values of the validation set samples was 0.881, with a standard error prediction (SEP) of 0.206. For starch, the RSQ between the measured and predicted values in the calibration set was 0.951, with an SEC of 1.523. The cross-validation 1-VR was 0.936, with an SECV of 1.751. The R² between the predicted values and the chemically measured values of the validation set samples was 0.923, with an SEP of 1.973 (Table 1). The NIRS detection models established in this study demonstrated favorable prediction accuracy and stability, and could meet the requirements for rapid and non-destructive determination of sorghum.

**Table 1:** Evaluation parameters of sorghum tannin and starch content models in calibration and validation set

	Calibration set							Validation set						
Traits	Samples	Range	Mean	SEC	RSQ	SECV	1-VR	Traits	Samples	Range	Mean	SEP	R^2^	RPD
Tannin	126	0.01–2.73	0.813	0.191	0.916	0.201	0.894	Tannin	30	0.04–2.66	0.802	0.206	0.881	2.954
Starch	126	56.07–82.36	69.96	1.523	0.951	1.751	0.936	Starch	30	57.69–80.28	68.79	1.973	0.923	2.898

*SEC* standard error of calibration, *SECV* standard error of cross validation, *RSQ* coefficient of determination in calibration, *1-VR* coefficient of determination in cross validation, *SEP* standard error prediction, *R*^2^ coefficient of determination, *RPD* residual prediction deviation

### Phenotypic diversity analysis

In order to evaluate the phenotypic variations of liquor-brewing quality related traits in the GWAS population with 214 sorghum cultivars, the tannin and starch contents were determined by NIRS. The mean values, ranges, coefficients of variation (CV) and broad-sense heritability (*H*^*2*^) for these traits were shown in Table 2. Great differences of the CV in all environments were found for the two traits. Overall, the sorghum cultivars in this GWAS panel clearly exhibited considerable natural variations in the two liquor-brewing quality related traits and displayed very high genetic diversity. BLUPs for the two liquor-brewing quality related traits were predicted based on two years and two locations of replicated phenotyping data. Tannin content exhibited a relatively high heritability of 73.4%, indicating that phenotypic variation in this trait is predominantly governed by a few major-effect genes with relatively minor environmental influence. In contrast, starch content displayed a moderate heritability of 50.59%, suggesting that this trait is more susceptible to environmental fluctuations and likely controlled by a combination of genetic and environmental factors. We analyzed the characteristics of tannin and starch contents in sorghum from different ecological regions based on the BLUPs. Overall, sorghum accessions from countries with a long‑standing tradition of liquor brewing (e.g., China, Japan, and Russia) exhibited significantly higher tannin content than those from other regions (Supplementary Fig. S1a). In contrast, accessions from India, where sorghum is predominantly used for food, and from the United States, where it is mainly used for feed, showed significantly higher starch content compared to accessions from the other countries (Supplementary Fig. S1b).

**Table 2:** Phenotypic statistics of tannin and starch content of sorghum

Trait	Environment	Max	Min	Average	CV/%	Skewness	Kurtosis	H^2^
Tannin content	NJ2023	2.73	0.01	0.81	85.02	0.52	-0.74	73.40%
	NJ2024	2.92	0.01	0.81	82.11	0.67	-0.32	
	YC2024	2.58	0.01	0.84	79.94	0.62	-0.73	
	BLUPs	2.74	0.01	0.81	75.57	0.76	0.16	
Starch content	NJ2023	82.36	56.07	69.30	9.66	0.084	-0.94	50.59%
	NJ2024	81.73	55.06	68.85	10.95	-0.15	-0.96	
	YC2024	81.70	58.39	70.54	7.75	-0.42	-0.54	
	BLUPs	79.83	56.38	69.48	8.08	-0.19	-0.41	

NJ2023: The environments of Nanjing in 2023; NJ2024: The environments of Nanjing in 2024; YC2024: The environments of Yuci in 2024

H^2^: Broad-sense heritability

## Population structure, kinship and LD decay analysis

A total of 545,350 SNP markers were retained after filtering. Based on these SNP markers, population structure analysis and kinship analysis were performed. Using the ADMIXTURE software, the cross-validation error was calculated, and the population was divided into nine subpopulations. Kinship analysis revealed distant phylogenetic relationships among most varieties. PCA analysis showed almost no varieties with the same background. These results demonstrated that the selected population was suitable for GWAS and could be used to identify the number of variants in specific loci. To estimate the LD level of the population, the r² value was calculated using PLINK software. The LD decay distance ranged from 9.9 to 70.2 kb, with an average of 17.4 kb [22].

### GWAS for sorghum tannin and starch contents

To investigate the genetic basis of natural variations in sorghum grain tannin and starch contents, we conducted a GWAS with the mixed linear models (MLMs) simultaneously accounting for population structure and relative kinship matrix based the collection of phenotype data from three environments. As results, a total of eighteen associations with 338 significant SNPs which exceeded the threshold were observed (Supplementary Table S2).

**Fig. 1:**
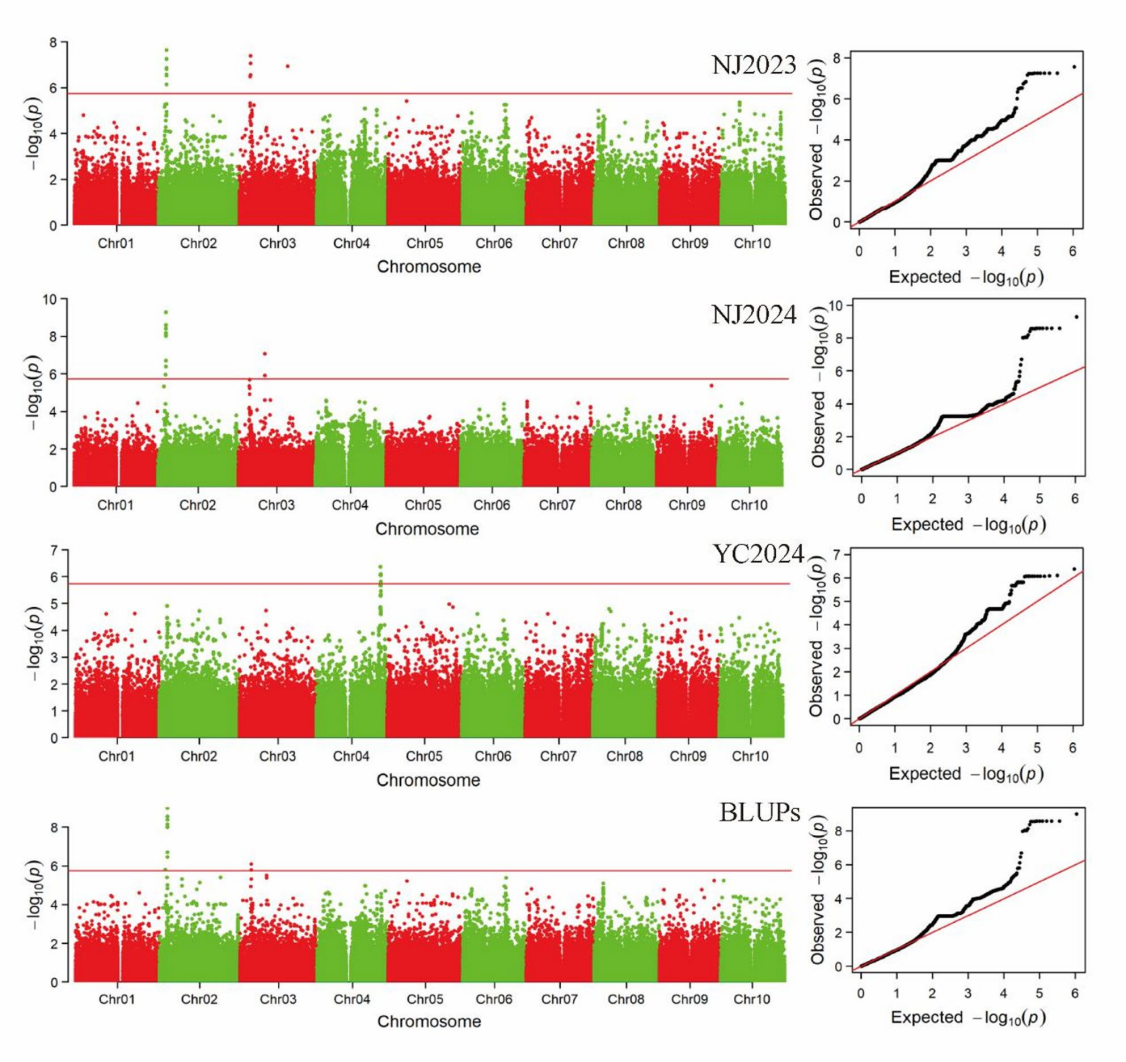
Manhattan and Q–Q plots for tannin content in the environments of Nanjing in 2023, 2024, Yuci in 2024 and BLUPs. The horizontal dotted lines of the Manhattan plots with red color represent the genome-wide significance threshold

A total of five associations with 67 SNPs significantly associated with tannin content were detected on three chromosomes (Chr02, Chr03 and Chr04) (Fig. 1 and Supplementary Table S2). In the environment of Nanjing in 2023, two associations with 23 SNPs significantly associated with tannin content were detected, with significant peaks observed at Chr02_7982502 and Chr03_10847028, respectively. In the environment of Nanjing in 2024, two associations with 22 SNPs were detected, with significant peaks observed at Chr02_7982502 and Chr03_25875862. In the environment of Yuci in 2024, only one association with 22 SNPs was detected, with the significant peak observed at Chr04_62083103.

**Fig. 2:**
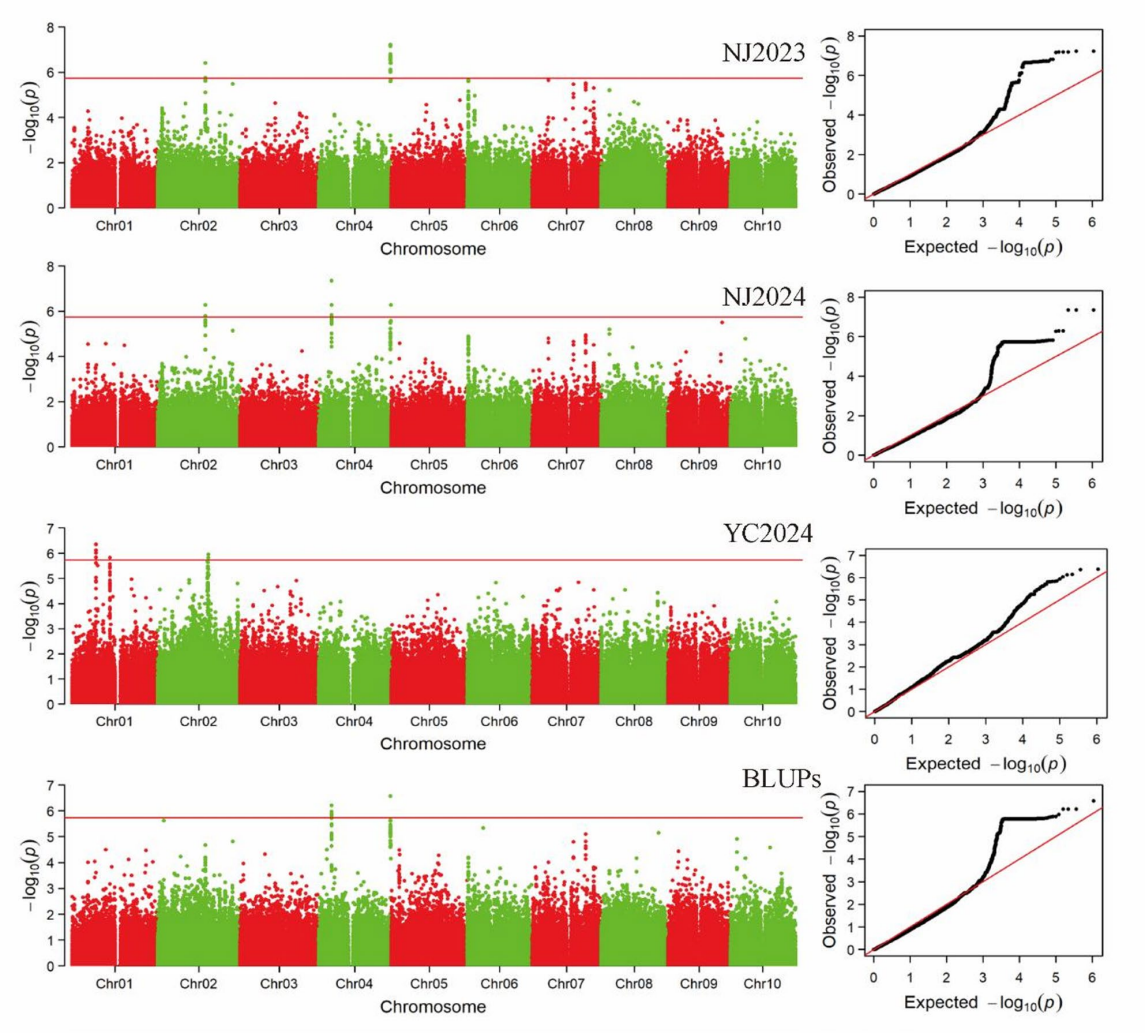
Manhattan and Q–Q plots for starch content in the environments of Nanjing in 2023, 2024, Yuci in 2024 and BLUPs. The horizontal dotted lines of the Manhattan plots with red color represent the genome-wide significance threshold

A total of eight associations with 87 SNPs significantly associated with starch content were detected on three chromosomes (Chr01, Chr02 and Chr04) (Fig. 2 and Supplementary Table S2). In the environment of Nanjing in 2023, two associations with 57 SNPs significantly associated with starch content were detected, with significant peaks at Chr02_45158091 and Chr04_68016029. In the environment of Nanjing in 2024, three associations with 19 SNPs were detected, with significant peaks at Chr02_45158616, Chr04_68112330 and Chr04_12163865. In the environment of Yuci in 2024, three associations with 11 SNPs were detected, with significant peaks at Chr01_22782600, Chr01_35968421 and Chr02_47725537.

A total of five associations with 184 SNPs significantly associated with tannin and starch contents were detected based on the BLUPs (Figs. 1 and 2 and Supplementary Table S2). Three associations with 23 SNPs significantly associated with tannin content were detected, with significant peaks observed at Chr02_5792036, Chr02_7982502 and Chr03_10847028, respectively. Two associations with 161 SNPs significantly associated with starch content were detected, with significant peaks observed at Chr04_12163865 and Chr04_68014847.

### Stably inherited QTL Analysis

The GWAS association signals detected across multiple environments suggest that they are likely stably inherited QTLs. For sorghum tannin content, the associations with significant peaks at Chr02_7982502 (Nanjing 2023, Nanjing 2024 and BLUPs), represent the same genetic locus associated with phenotypic signals across different years, and were detectable in different environments. This locus is proximal to the reported *Tan2* gene (Sobic.002G076600), which encodes a bHLH transcription factor regulating proanthocyanidin biosynthesis. The association with significant peak at Chr03_10847028 was detected significantly associated with tannin content in Nanjing in 2023 and BLUPs. Detectable associations across different environments were also present for starch content. The associations with significant peaks at Chr02_45158091 (Nanjing 2023) and Chr02_45158616 (Nanjing 2024) with a physical distance of less than 1 kb apart were considered to represent the same locus. The association with a significant peak at Chr04_68112330 detected in the 2024 Nanjing environment also exceeded the threshold in the 2023 Nanjing environment and BLUPs.

### Haplotype analysis

**Fig. 3:**
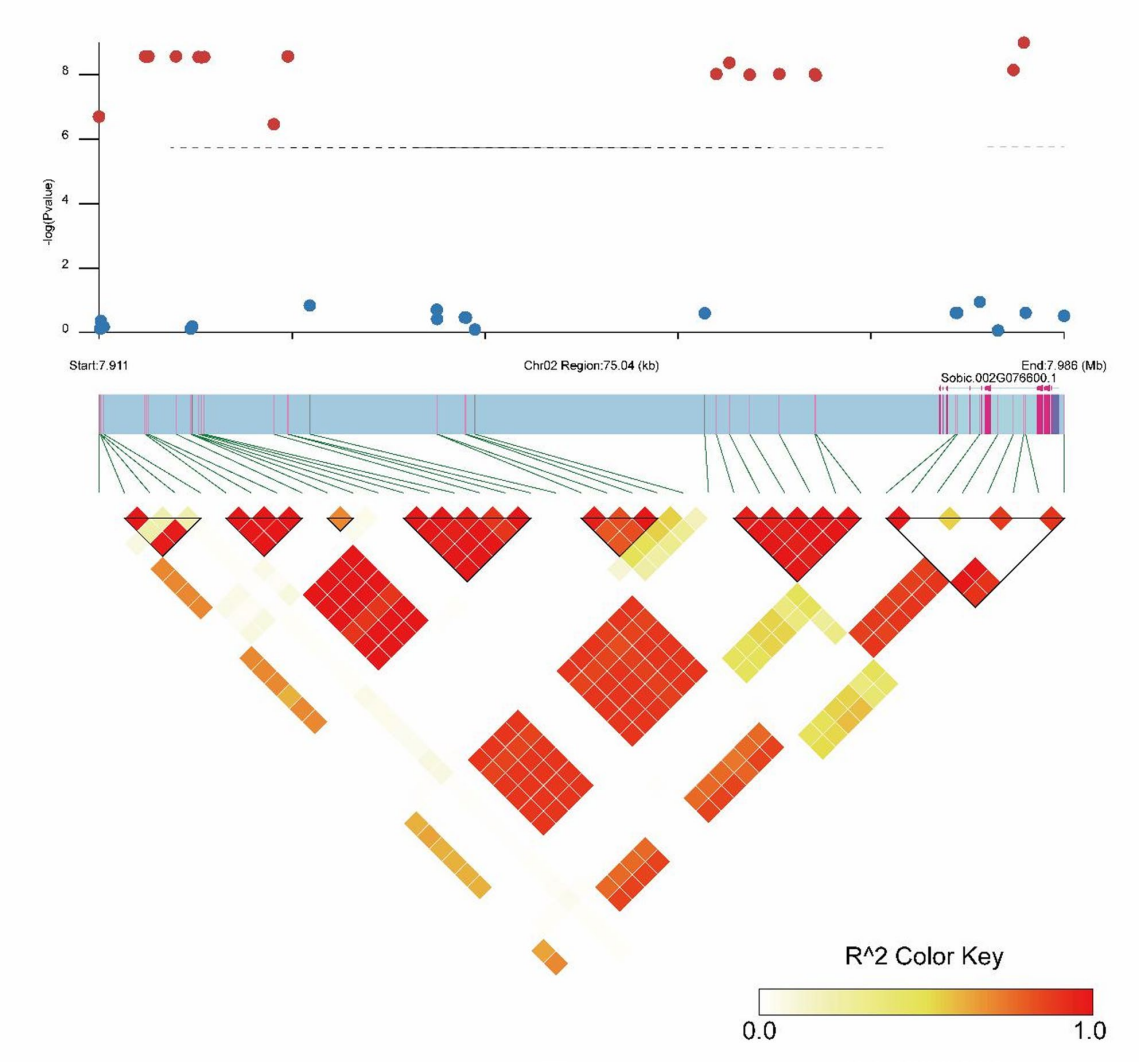
Local Manhattan plot of the sorghum *Tan2* gene and linkage disequilibrium heat map. The horizontal dashed line represents the genome-wide significance threshold

**Fig. 4:**
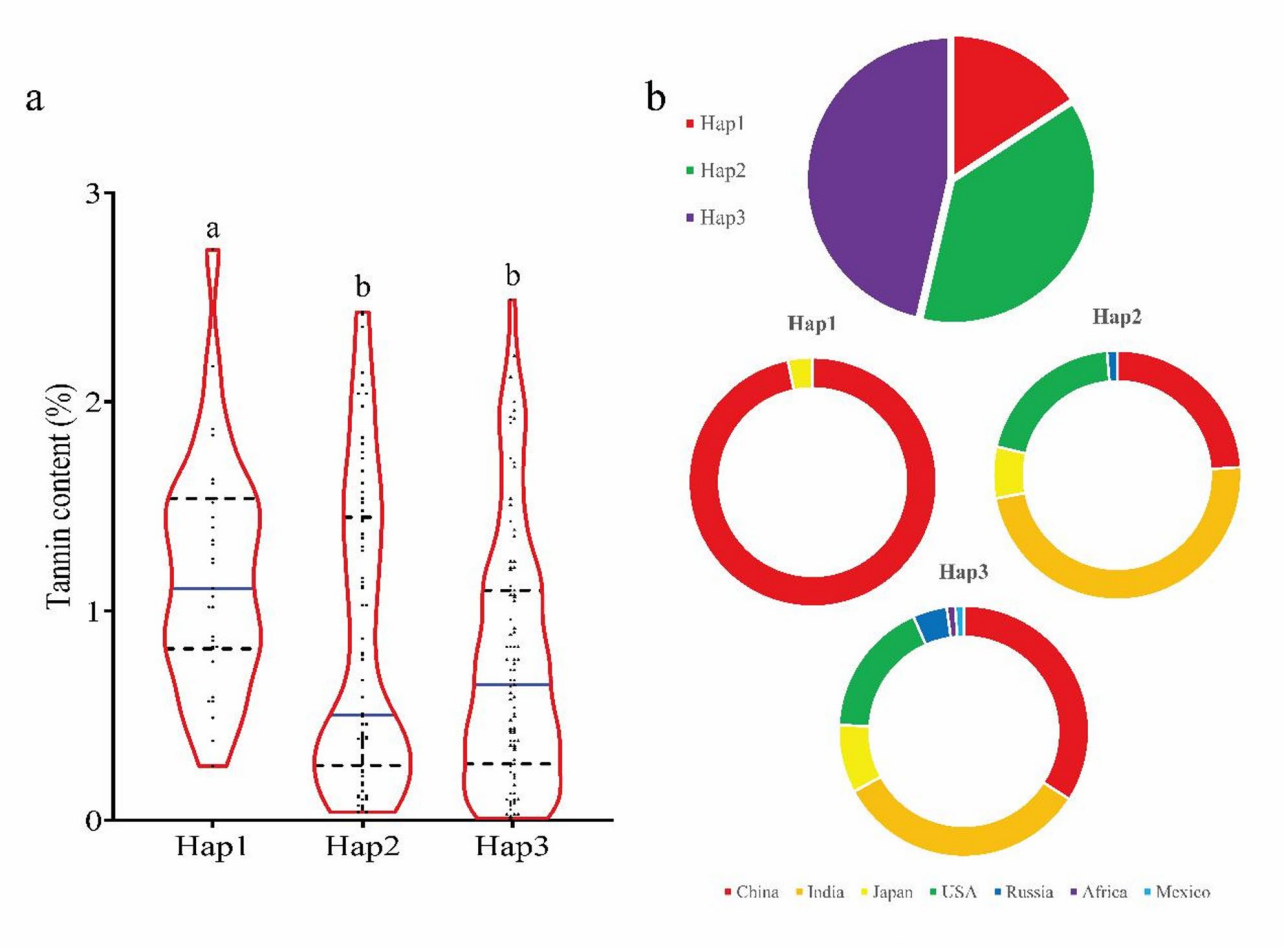
The haplotype analysis of the sorghum *Tan2* gene. (**a**) Statistical analysis of tannin content variations across distinct haplotypes. Different letters represent significant differences (*P* < 0.05) using Duncan`s multiple range test. (**b**) The geographical distribution of different haplotypes

The sorghum *Tan2* gene (Sobic.002G076600) was the causal genes for tannin content on Chr02 according to the LD block analysis (Fig. 3). Haplotype analysis was performed to investigate the genes mutation of *Tan2* in sorghum (Fig. 4). The sorghum *Tan2* gene harbored five mutation sites, including two nonsynonymous SNP mutations. A T/C SNP at the position of 7,979,912 bp caused an amino acid substitution from tryptophan (Trp) to arginine (Arg) and a T/G SNP at the position of 7,983,795 bp led to a substitution from histidine (His) to glutamine (Gln). Three haplotypes (hap1, hap2 and hap3) were defined based on *Tan2* gene SNP variations (Supplementary Table S3). The haplotype analysis showed that hap1 was superior compared to the other two based on the tannin data. In all varieties, hap1 showed the lowest proportion in current germplasm resources, indicating this elite haplotype remained underutilized. Combined with geographic origin tracing, the dominant haplotype hap1 was most prevalent in Chinese germplasm. This suggested that the *Tan2* candidate gene exhibits higher polymorphism within the Chinese sorghum population, providing critical resources for future molecular marker-assisted breeding.

**Fig. 5:**
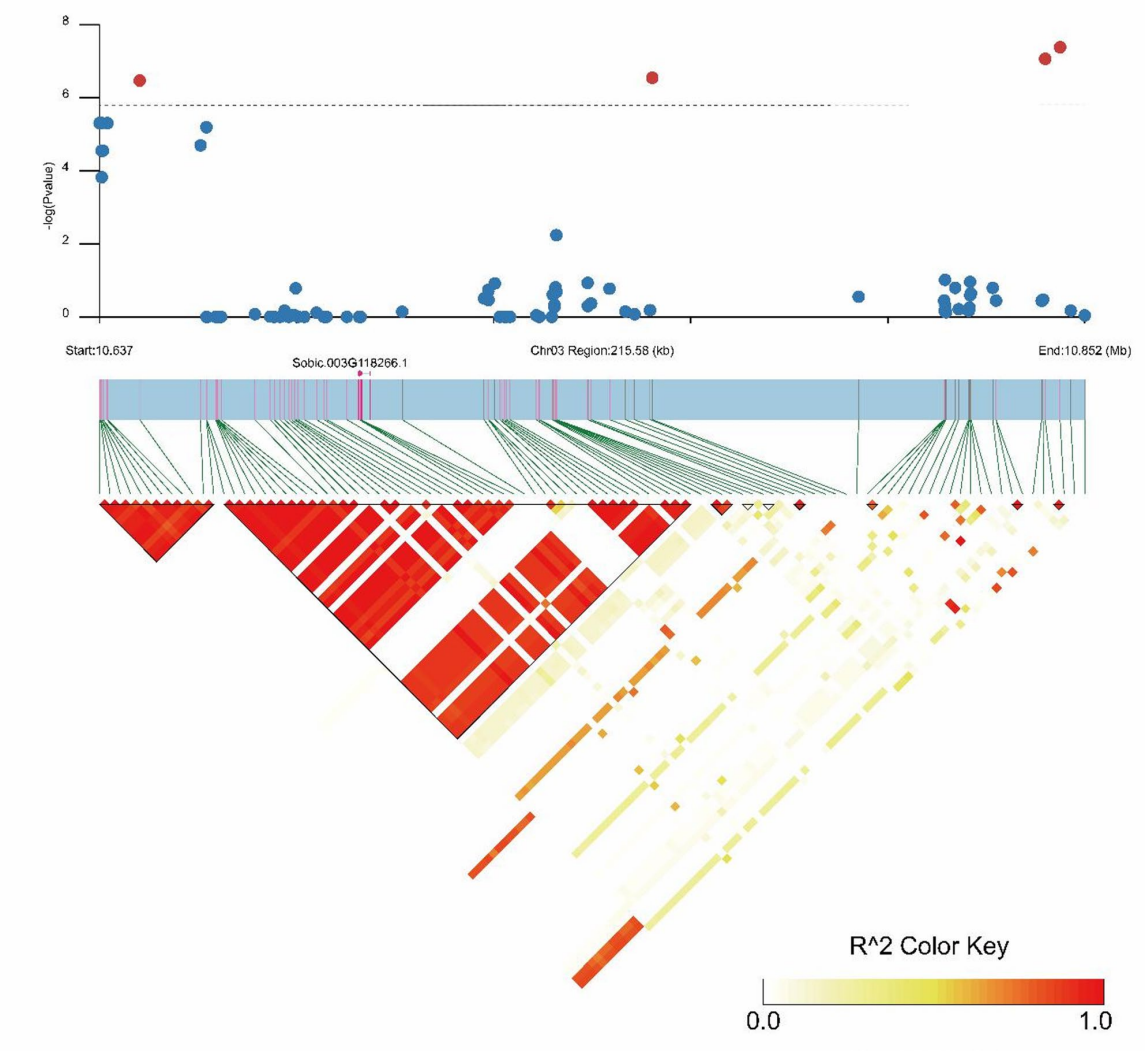
Local Manhattan plot of Sobic.003G118266 and linkage disequilibrium heat map. The horizontal dashed line represents the genome-wide significance threshold

**Fig. 6:**
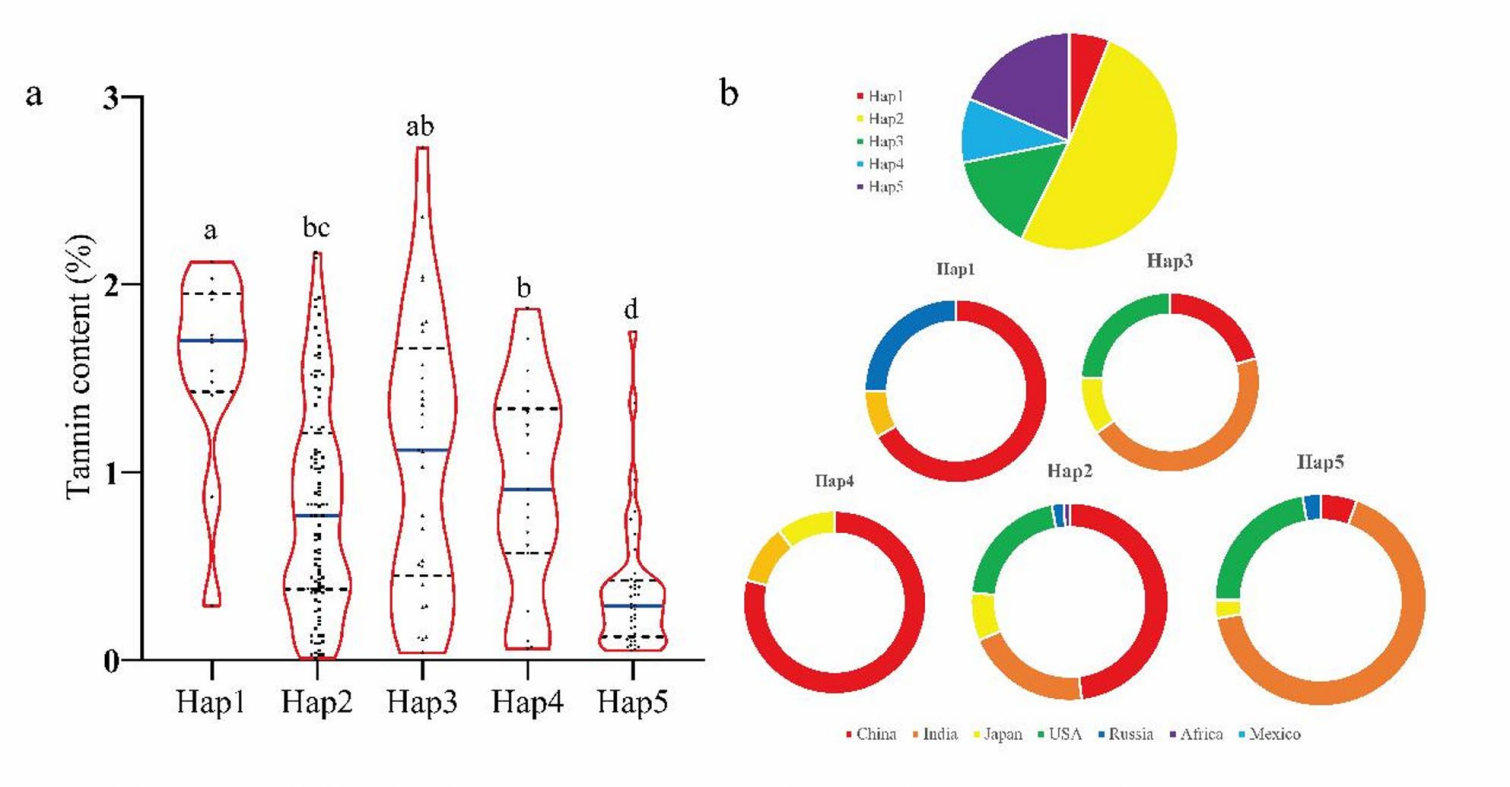
The haplotype analysis of the gene Sobic.003G118266. (**a**) Statistical analysis of tannin content variations across distinct haplotypes. Different letters represent significant differences (*P* < 0.05) using Duncan`s multiple range test. (**b**) The geographical distribution of different haplotypes

To identify the causal genes in the QTL for tannin content on Chr03, we calculated the LD block. The significant SNP was in the LD block from 10,636,869 to 10,852,447 (Fig. 5). In the LD blocks, there were 23 predicted genes (Supplementary Table S6), of which the Sobic.003G118266 was annotated as chalcone synthase (CHS), a key enzyme involved in the biosynthesis of flavonoids. We performed haplotype analysis to investigate the gene mutations of Sobic.003G118266 (Fig. 6). The sorghum gene Sobic.003G118266 harbored seven nonsynonymous SNP mutation sites. A total of five haplotypes (hap1, hap2, hap3, hap4 and hap5) were defined based on SNP variations in the gene region (Supplementary Table S4). The superior haplotype hap1 provided the least percentages compared with the other four haplotypes, suggesting that the elite haplotype was not fully used. The dominant haplotype hap1 was most prevalent in Chinese germplasm. Germplasm carrying hap5 exhibited the lowest tannin content, and the accessions of hap5 were mainly from India. Generally, the tannin concentration in sorghum cultivars is related to grain color [34]. Throughout breeding history, breeders have primarily relied on grain color rather than tannin content to develop high-tannin sorghum through conventional breeding. We downloaded transcriptome data of sorghum with different grain colors from NCBI (PRJNA1255307) and analyzed differentially expressed genes. The expression level of the gene Sobic.003G118266 was highest in black grains and lowest in white grains.

**Fig. 7:**
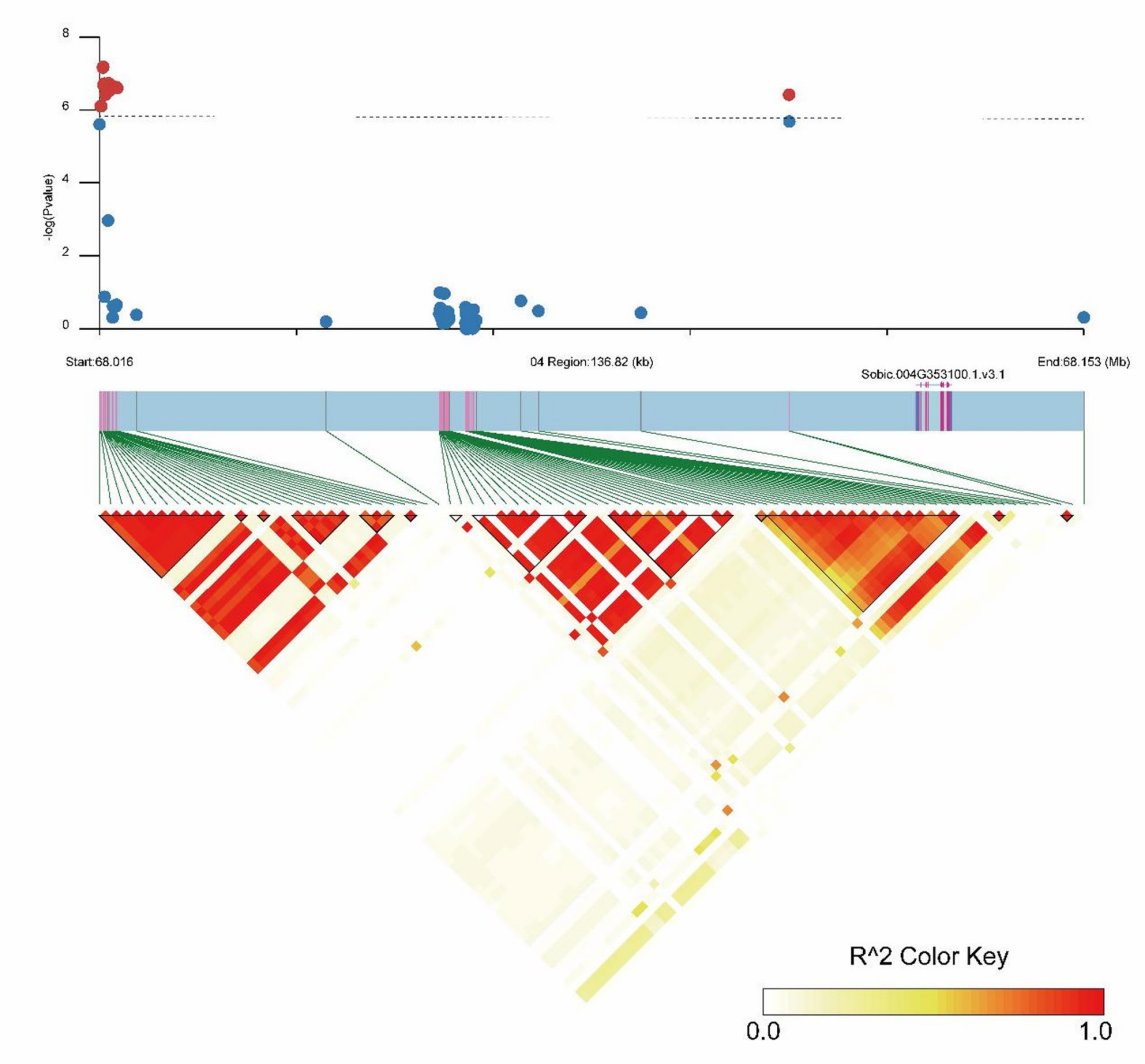
Local Manhattan plot of Sobic.004G353100 and linkage disequilibrium heat map. The horizontal dashed line represents the genome-wide significance threshold

**Fig. 8:**
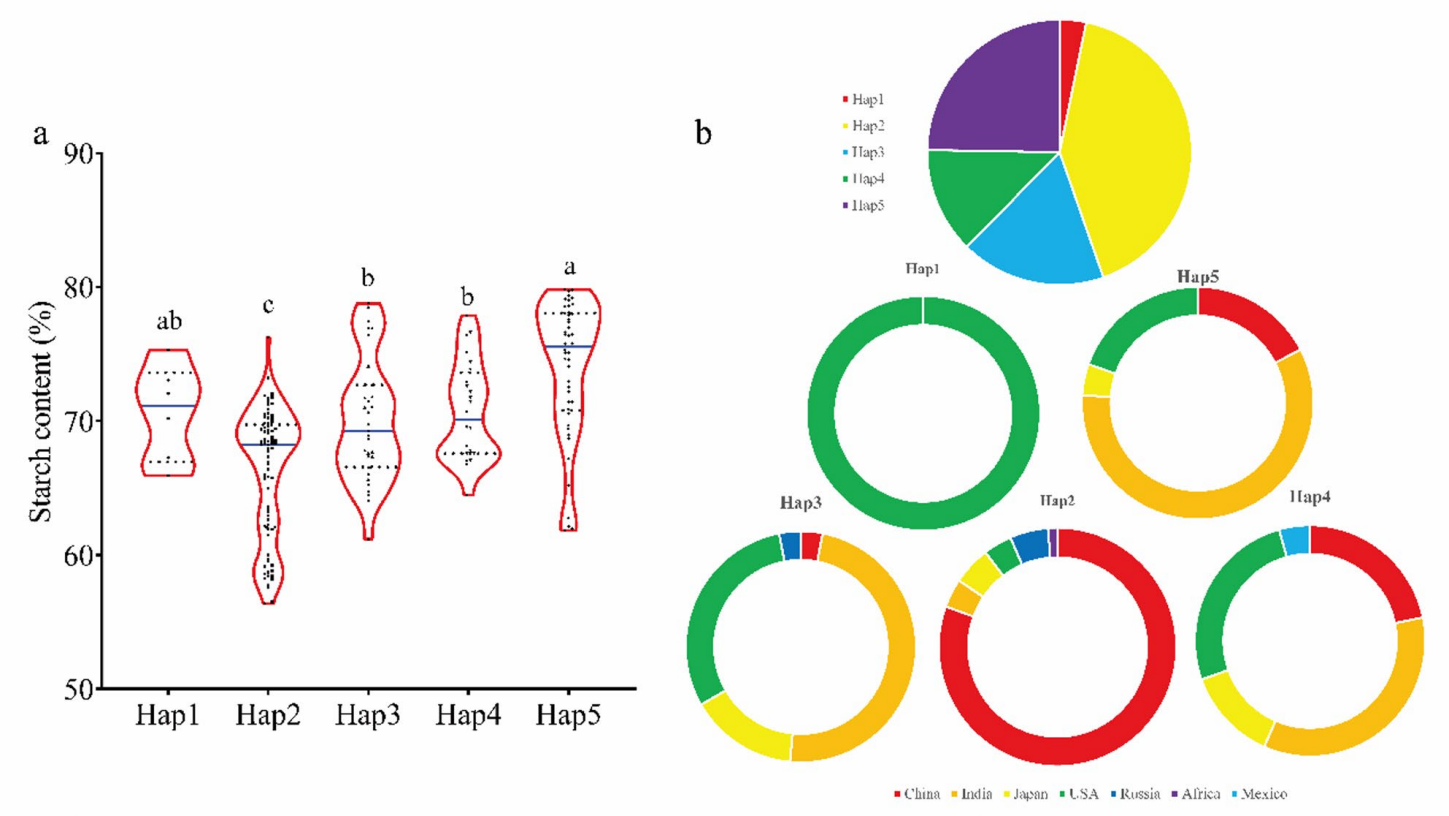
The haplotype analysis of the gene Sobic.004G353100. (**a**) Statistical analysis of starch content variations across distinct haplotypes. Different letters represent significant differences (*P* < 0.05) using Duncan`s multiple range test. (**b**) The geographical distribution of different haplotypes

To identify the causal genes in the QTL for starch content on Chr04, we calculated the LD block. The significant SNP was in the LD block from 68,016,493 to 68,153,310 (Fig. 7). In the LD blocks, there were 21 predicted genes (Supplementary Table S7), of which the Sobic.004G353100 was annotated as glucose-6-phosphate translocator (G6PT), which is a key regulatory node in plant starch synthesis. So, the Sobic.004G353100 gene was mostly the candidate gene. To investigate the gene mutations of Sobic.004G353100, we performed haplotype analysis (Fig. 8). The sorghum gene Sobic.004G353100 harbored two mutation sites, including only one nonsynonymous SNP mutations: A G/A SNP at the position of 68,133,678 bp caused an amino acid substitution from alanine (Ala) to valine (Val). We extracted the promoter sequence of Sobic.004G353100 and analyzed the variation sites within the promoter region. A total of five haplotypes (hap1, hap2, hap3, hap4 and hap5) were defined based on SNP variations in the promoter and gene region (Supplementary Table S5). The haplotype analysis showed that hap5 was superior compared to the others based on the starch data. Combined with geographic origin tracing, the accessions of hap5 almost spread all over the regions where we collected the germplasm and the dominant haplotype hap5 exhibits the highest frequency in Indian germplasm resources. Germplasm carrying hap5 exhibits the highest starch content, while that with hap2 showed the lowest. The accessions of hap2 were mainly from China, suggesting that the candidate gene Sobic.004G353100 exhibits considerable natural variations and displayed very high genetic diversity in Chinese sorghum germplasm.

## Discussion

Tannin and starch contents in sorghum are crucial indicators for evaluating its brewing quality. Although these parameters can be determined using chemical methods, such methods are generally time-consuming, labor-intensive, destructive to seeds, costly, and involve complex detection procedures. Particularly in the genetic improvement of sorghum brewing quality, it is difficult to meet the demand for rapid, non-destructive testing of large sample volumes. In contrast, near-infrared rapid detection technology offers advantages such as high efficiency, simple operation, and non-destructive testing of seeds. Establishing a near-infrared model for non-destructive determination of sorghum brewing quality content enables rapid and accurate analysis, serving as an essential prerequisite for improving sorghum brewing quality. Currently, research on the application of near-infrared technology in sorghum quality assessment is relatively limited [35], which significantly hinders the study of the genetic basis of sorghum liquor-brewing quality traits. The NIRS detection models established in this study demonstrated favorable prediction accuracy and stability, and enabled high-throughput, multi-environment phenotyping, thereby improving statistical power and trait stability in GWAS. Regarding the model’s predictive performance, the RPD values both for tannin and starch reached close to 3.0. According to the widely accepted criteria [36, 37], these values met the requirements for rapid detection and further supported the reliability of our NIRS-based phenotyping approach, although the sample size (*n* = 156) used for constructing the NIRS models might limit the predictive accuracy to some extent. Future work will focus on collecting additional samples from a broader genetic background to further improve model accuracy.

Sorghum, as one of the major cereal crops, possesses existing germplasm resources with broad genetic and phenotypic variation. This study focused on analyzing the variation characteristics of tannin and starch contents, finding that both exhibited significant variations within the association panel, indicating the complexity of their respective biosynthetic processes. The variations in these quality traits may provide new resources for sorghum breeding. Human selection for different end-uses influences the distribution patterns of quality traits in germplasm resources [6]. For instance, in this association panel, sorghum accessions from China, Japan, and Russia showed the highest tannin content. These three countries are major producers of distilled spirits. China’s Baijiu is world-renowned, while Japan’s Sake and Russia’s Vodka also have significant global influence. The previously reported predominant hap1 of the *Tan2* gene was also mainly distributed in China and Japan. The predominant hap1 of the gene Sobic.003G118266, encoding CHS, was mainly distributed in China and Russia. These materials were expected to become a genetic resource bank for tannin improvement in sorghum. India, as a populous developing country, is one of the world’s largest sorghum producers. Sorghum is an important staple crop for hundreds of millions of people, especially in the arid and semi-arid regions of the central and western parts of the country. Sorghum accessions from India exhibited the highest starch content and lowest tannin content. The predominant hap5 of the gene Sobic.004G353100, encoding G6PT, and the recessive hap5 of the gene Sobic.003G118266, annotated as CHS, were mainly distributed in India. These distribution patterns may reflect long-term selection pressures associated with regional end-use preferences, although historical germplasm exchange and breeding practices may also contribute.

The presence of genotype × genotype (G×G) and genotype × environment (G×E) interactions makes the identification of stable QTLs across diverse genetic backgrounds and environmental conditions the most challenging aspect of QTL studies [38]. Consequently, most QTLs exhibit considerable environmental sensitivity, leading to inconsistent mapping results under different environmental scenarios [39]. In this study, a total of eighteen significant associations scattered over 4 chromosomes were detected for tannin and starch contents. Among these, the associations on Chr02 and Chr03 for tannin content, Chr02 and Chr04 for starch content were expressed in different environments, indicating that they were likely to be stable QTLs. These results provided important genetic variations for accelerating the improvement of liquor-brewing quality related traits in sorghum. The stable QTLs associated with tannin and starch contents described above represents ideal targets for marker-assisted selection (MAS) in sorghum breeding programs. The superior haplotypes identified within these stable QTL regions could be effectively employed to improve grain quality for liquor production. Furthermore, we will prioritize further functional validation of these stable QTLs to accelerate the development of elite sorghum varieties with optimized tannin and high starch contents.

Interestingly, stable QTLs were exclusively detected in the environment of Nanjing across different years, whereas no co-expressed QTL were observed in Yuci. These results indicated that sorghum germplasm exhibits strong regional adaptability, and that differences between ecological regions exert a greater impact on sorghum tannin and starch contents than annual variations. Such environmental adaptability may contribute to variations in grain quality traits. Temperature conditions differ markedly between Nanjing (southern China) and Yuci (northern China), particularly during the grain-filling period. High-temperature stress during the grain-filling stage has been shown to severely inhibit starch biosynthesis, with significant alterations in the temporal expression profiles of genes involved in sugar cleavage, utilization, transport, and starch biosynthesis in heat-stressed plants [40]. The effect of high temperature on tannin synthesis is complex and species-specific, generally suppressing the accumulation of precursor substances and thereby reducing tannin synthesis [41]. Differences in rainfall patterns between the two locations also affect the brewing quality of sorghum. Precipitation is the primary environmental factor driving starch biosynthesis and structural assembly, while drought stress significantly reduces starch accumulation [42]. In addition, the synthesis and accumulation of tannins represent key adaptive strategies of plants to drought environments [43]. The absence of co-expressed QTLs in Yuci may therefore reflect site-specific climatic or edaphic factors, underscoring the importance of incorporating diverse agro-ecological zones in future studies.

Three nonsynonymous SNP variations revealed seven haplotypes of the *Tan2* gene in previous studies [7, 10, 13]. A T/C SNP at the position of 7,979,912 bp caused an amino acid substitution from tryptophan (Trp) to arginine (Arg). A T/G SNP at the position of 7,983,795 bp resulted in a substitution from histidine (His) to glutamine (Gln). A C/T SNP at the position of 7,983,859 bp led to premature transcription termination. In this study, haplotype analysis detected only two nonsynonymous SNP mutations, while the C/T SNP at the position of 7,983,859 bp which caused premature termination of the *Tan2* protein was not identified. This discrepancy was attributed to the MAF of the C/T SNP at the position of 7,983,859 bp being less than 0.05 in the sorghum diversity panel resulting in no significant signal detected in GWAS.

Genes related to anthocyanin biosynthesis can be categorized into two classes: structural genes, which encode functional enzymes catalyzing biosynthetic reactions, and regulatory genes, primarily encoding transcription factors that control the expression of structural genes. To date, only four regulatory genes involved in tannin biosynthesis have been identified in sorghum. Tannin1 (*Tan1*, Sobic.004G280800) regulates the synthesis of anthocyanins and proanthocyanidins by encoding a WD40-repeat protein, which is the ortholog of TRANSPARENT TESTA GLABRA1 (TTG1), in Arabidopsis, thereby influencing grain nutritional quality [10]. Interestingly, in this study, the association signal detected in Yuci environment in 2024 was located very close to the Tannin1 gene, less than 200 kb away. Given that the marker density in this region was much lower than the average marker density on chr04, and considering that there was a gap in this region, increasing the marker density would allow us to detect the Tannin1 gene locus. Tannin2 (*Tan2*, Sobic.002G076600) encodes a protein containing a bHLH domain and corresponds to the B1 locus gene. This gene is orthologous to *TT8* (in Arabidopsis), *Rc* (in rice), and *IN1* (in maize), all involved in regulating the phenylpropanoid biosynthetic pathway [13]. Furthermore, researchers have cloned the MYB transcription factor (*Y1*, Sobic.001G398100), which governs both pericarp pigmentation and the accumulation of 3-deoxyanthocyanidins in the sorghum pericarp [14]. Its non-functional allele results in the absence of certain pigments in the pericarp due to a large genomic deletion [44]. Another key R2R3-MYB gene, *SbC1*, that specifically regulates anthocyanin accumulation in sorghum coleoptiles but not in grain was identified through GWAS, virus induced gene silencing (VIGS) experiment and haplotype analysis [16]. In this study, a promising structural gene Sobic.003G118266 which encodes CHS was identified. The key enzymes involved in anthocyanin biosynthesis primarily include chalcone synthase, Flavanone 3-hydroxylase, Flavonoid 3’-hydroxylase, and Leucoanthocyanidin reductase [45]. CHS, as pivotal enzymes in the phenylpropanoid pathway, exert dual regulatory effects on tannin dynamics through their differential modulation of specialized metabolic fluxes. The sorghum gene Sobic.003G118266 is an ortholog of the Arabidopsis *TT4* gene (AT5G13930), a key enzyme involved in the biosynthesis of flavonoids in Arabidopsis [46]. Elevating the expression level of the *TT4* gene significantly increases Arabidopsis anthocyanin content [47]. Although direct functional evidence in sorghum is lacking, the strong association signal and ortholog to TT4 suggest that Sobic.003G118266 is a promising candidate influencing tannin accumulation. We further suggest that future studies could employ approaches such as expression analysis across different developmental stages and tissues, VIGS, and transgenic complementation or knockout lines to elucidate the molecular mechanisms by which these genes contribute to grain quality traits.

The biosynthesis and assembly of starch in cereals are catalyzed by multiple key enzymes, including ADP-glucose pyro phosphorylase, soluble starch synthase, starch branching enzyme, starch debranching enzyme, and granule-bound starch synthase [48]. In sorghum, mutations in the waxy gene encoding granule-bound starch synthase result in kernels with little or no amylose content, thereby enhancing starch digestibility [49]. The Wx gene responsible for waxy starch encodes a granule-bound adenosine diphosphate glucosyltransferase. The digestibility of sorghum starch is regulated by distinct allelic variations in the Wx gene, including an insertion in the third exon (wx^a^), a missense mutation within a conserved domain (wx^b^), and a point mutation (wx^c^) [50, 51]. Additionally, researchers have characterized another type of sugary mutants that exhibit high levels of water-soluble carbohydrates in their endosperm [20]. In this study, a promising gene Sobic.004G353100 annotated as G6PT was identified. G6PT is a key regulatory node in plant starch synthesis. By regulating phosphate concentration and carbon substrate supply, it directly impacts the activity of AGPase and the production efficiency of ADP-glucose. ADP-glucose is then utilized by starch synthase and branching enzyme to form amylose and amylopectin of starch. Based on its annotated function and pathway position, Sobic.004G353100 represents a strong candidate gene; however, its direct role in regulating starch accumulation in sorghum grains requires functional validation.

The haplotype analysis showed that superior elite haplotype of *Tan2* and Sobic.003G118266 showed the lowest proportion in current germplasm resources, indicating these elite haplotypes remained underutilized. This conclusion is based on the assumption that low frequency results in underutilization. However, low frequency may be due to other reasons, such as the haplotype being linked to other unfavorable agronomic traits, or being excluded during breeding. Currently, there has been a report of precise co-localization between major chilling tolerance QTLs and the classical *Tan2* gene, as well as the dwarfing genes [52]. However, no studies have yet reported that *Tan2* directly leads to clear unfavorable traits such as reduced yield, smaller grain size, or decreased pest and disease resistance. The *Tan2* gene may have had its superior haplotype inadvertently excluded in modern breeding, partly because they did not align with the mainstream breeding objectives at the time, such as dwarfing and chilling tolerance. Additionally, we examined the distribution of superior haplotypes in existing germplasm resources. The superior haplotype *Tan2* hap1 is present in 33 accessions, 32 of which originated from China. Among the 28 accessions with clear geographic provenance, more than half are from landraces in Jiangsu. The other superior haplotype, Sobic.003G118266 hap1, comprises 13 accessions, nine from China and 3 from Russia; among these, the five Chinese accessions with clear geographic provenance are all landraces from Jiangsu. In recent years, due to low profitability, sorghum cultivation in Jiangsu has been reduced to scattered distribution. These superior haplotypes have gradually been eliminated with the reduction in Jiangsu’s sorghum cultivation area and are poorly represented in modern improved varieties, implying that they may have been unintentionally excluded during breeding. Next, landraces carrying the superior haplotypes will be incorporated into the core germplasm repository and prioritized for future breeding programs. Large populations will be constructed to break the linkage between these superior haplotypes and other adaptive traits, and these haplotypes will be introgressed into modern elite genetic backgrounds using MAS.

## Conclusion

In this study, NIRS non-destructive calibration models for determining sorghum tannin and starch contents was established based on phenotypic identification of 214 representative sorghum cultivars. A total of 18 associations with 338 SNPs were detected significantly associated with tannin and starch contents. Among these, two associations for tannin content and two associations for starch content were expressed in different environments. Through LD block and haplotype analysis, a promising gene, Sobic.003G118266, was identified as orthologous to structural genes in known tannin synthesis pathways; while another promising gene, Sobic.004G353100, was determined to be a putative regulatory node in plant starch synthesis. 

## Supplementary Information

Supplementary Material 1.

Supplementary Material 2.

## Data Availability

All data used in the current study are included in this published article or are available from the corresponding author on reasonable request. The resequencing data were uploaded in the NCBI database (PRJNA826146).

## Abbreviations

GWAS: Genome-wide association study
SNPs: Single nucleotide polymorphisms
NIRS: Near-infrared reflectance spectrometer
QTLs: Quantitative trait loci
CHS: Chalcone synthase
G6PT: Glucose-6-phosphate translocator
LD: Linkage disequilibrium
H^2^: Broad-sense heritability
DMF: Dimethylformamide
GOPOD: Glucose oxidase-peroxidase-4-aminoantipyrine
OD: optical density
SNV: Standard normal variate
MPLS: Modified partial least squares
BLUPs: Best linear unbiased predictions
PCA: Principal component analysis
MLMs: Mixed linear models
MAF: Minor allele frequency
SEC: standard error of calibration
SECV: Standard error of cross validation
SEP: Standard error prediction
RPD: Residual prediction deviation
CV: coefficients of variation
VIGS: Virus induced gene silencing
MAS: Marker-assisted selection
