# Supplementary figures and images for: Genome-wide association analysis reveals genetic variations and candidate genes associated with tannin and starch contents in sorghum
Source: BMC Genomics. 2026 May 4;27:537. doi: 10.1186/s12864-026-12914-5 (PMC13262094; doi:10.1186/s12864-026-12914-5)

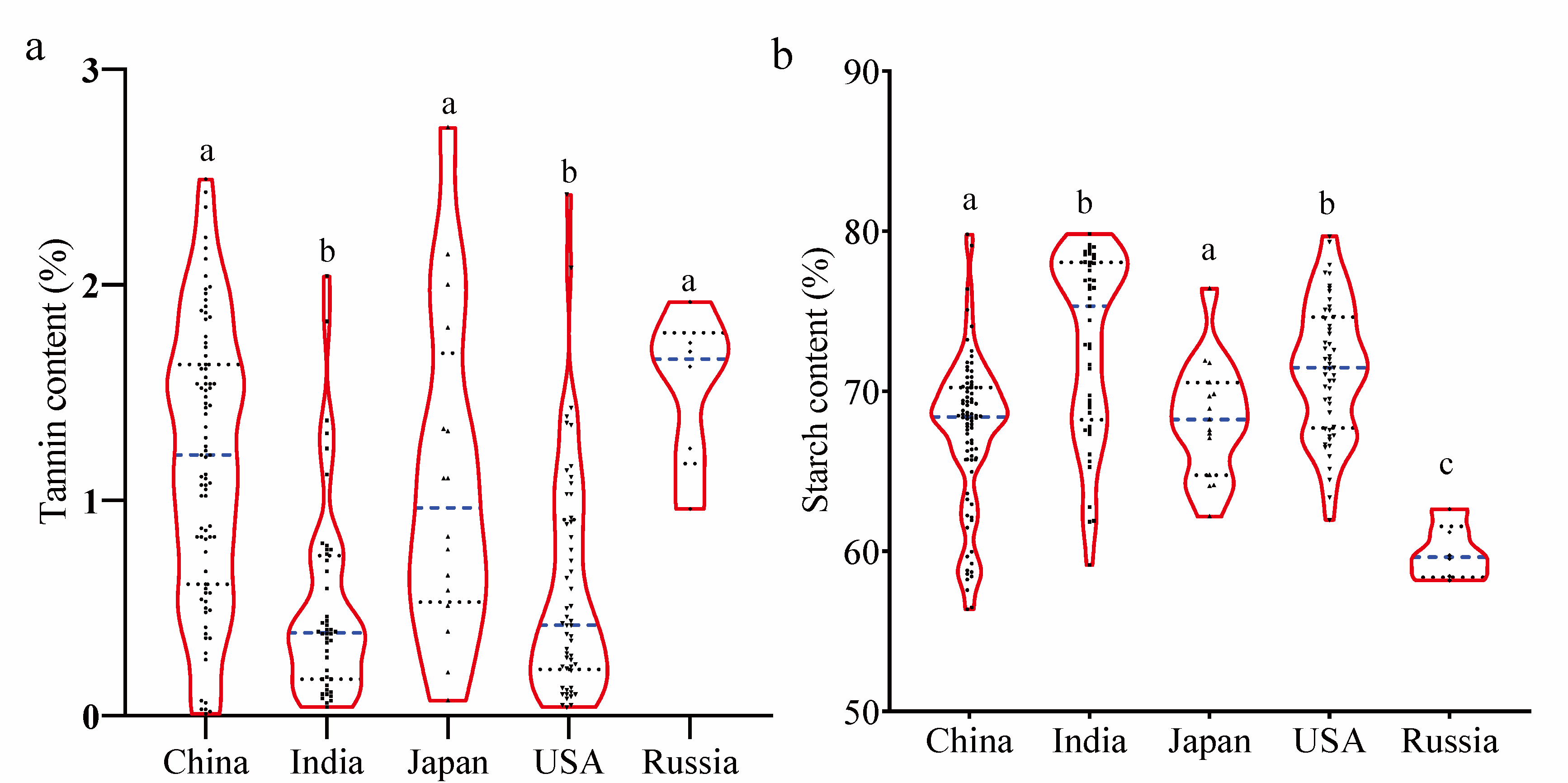

Supplement: Supplementary file 1 — Supplementary Material 1. [file 12864_2026_12914_MOESM1_ESM.tif]
