# Supplementary material for: Genome-wide association analysis reveals genetic variations and candidate genes associated with tannin and starch contents in sorghum
Source: BMC Genomics. 2026 May 4;27:537. doi: 10.1186/s12864-026-12914-5 (PMC13262094; doi:10.1186/s12864-026-12914-5)
Supplement: Supplementary file 2 — Supplementary Material 2. [file 12864_2026_12914_MOESM2_ESM.docx]

Table S1 The list of 214 sorghum cultivars used in this study.

| Variety name | Source | Providing institutions |
| --- | --- | --- |
| Saccaline | Japan | Sorghum Germplasm Resource Conservation Bank of Sorghum Research Institute of Shanxi Agricultural University |
| Double Grain Sorghum | China | Sorghum Germplasm Resource Conservation Bank of Sorghum Research Institute of Shanxi Agricultural University |
| Dval | Japan | Sorghum Germplasm Resource Conservation Bank of Sorghum Research Institute of Shanxi Agricultural University |
| Black Hull Sorghum | China | Sorghum Germplasm Resource Conservation Bank of Sorghum Research Institute of Shanxi Agricultural University |
| IS 1159C | India | Sorghum Germplasm Resource Conservation Bank of Sorghum Research Institute of Shanxi Agricultural University |
| Jinliang No.5 | China | Sorghum Germplasm Resource Conservation Bank of Sorghum Research Institute of Shanxi Agricultural University |
| Chaoxian Sugar Sorghum | China | Sorghum Germplasm Resource Conservation Bank of Sorghum Research Institute of Shanxi Agricultural University |
| CAHJ148 | China | Sorghum Germplasm Resource Conservation Bank of Sorghum Research Institute of Shanxi Agricultural University |
| Early Hegari SA #281 | U.S.A | Sorghum Germplasm Resource Conservation Bank of Sorghum Research Institute of Shanxi Agricultural University |
| Tx3197B | U.S.A | Sorghum Germplasm Resource Conservation Bank of Sorghum Research Institute of Shanxi Agricultural University |
| Cock Feterita | U.S.A | Sorghum Germplasm Resource Conservation Bank of Sorghum Research Institute of Shanxi Agricultural University |
| IS 9308 | India | Sorghum Germplasm Resource Conservation Bank of Sorghum Research Institute of Shanxi Agricultural University |
| Shunyi Yellow Sticky | China | Sorghum Germplasm Resource Conservation Bank of Sorghum Research Institute of Shanxi Agricultural University |
| Early Red Kafir #866 | U.S.A | Sorghum Germplasm Resource Conservation Bank of Sorghum Research Institute of Shanxi Agricultural University |
| Zhenzhubai | China | Sorghum Germplasm Resource Conservation Bank of Sorghum Research Institute of Shanxi Agricultural University |
| Sugar Sorghum3# | Japan | Sorghum Germplasm Resource Conservation Bank of Sorghum Research Institute of Shanxi Agricultural University |
| KS153-1 | U.S.A | Sorghum Germplasm Resource Conservation Bank of Sorghum Research Institute of Shanxi Agricultural University |
| LR-226 | U.S.A | Sorghum Germplasm Resource Conservation Bank of Sorghum Research Institute of Shanxi Agricultural University |
| Beiping No.7 | China | Sorghum Germplasm Resource Conservation Bank of Sorghum Research Institute of Shanxi Agricultural University |
| Black Amber # 7308 | U.S.A | Sorghum Germplasm Resource Conservation Bank of Sorghum Research Institute of Shanxi Agricultural University |
| Combine Kafir 60-1 | U.S.A | Sorghum Germplasm Resource Conservation Bank of Sorghum Research Institute of Shanxi Agricultural University |
| IS 4884C | India | Sorghum Germplasm Resource Conservation Bank of Sorghum Research Institute of Shanxi Agricultural University |
| A16023-1 | India | Sorghum Germplasm Resource Conservation Bank of Sorghum Research Institute of Shanxi Agricultural University |
| ICSH 25 | India | Sorghum Germplasm Resource Conservation Bank of Sorghum Research Institute of Shanxi Agricultural University |
| 02-512 | China | Sorghum Germplasm Resource Conservation Bank of Sorghum Research Institute of Shanxi Agricultural University |
| Fengtai White Sticky | China | Sorghum Germplasm Resource Conservation Bank of Sorghum Research Institute of Shanxi Agricultural University |
| Mays Amber | Japan | Sorghum Germplasm Resource Conservation Bank of Sorghum Research Institute of Shanxi Agricultural University |
| Dali 105 | China | Sorghum Germplasm Resource Conservation Bank of Sorghum Research Institute of Shanxi Agricultural University |
| Kaferita # 812 | U.S.A | Sorghum Germplasm Resource Conservation Bank of Sorghum Research Institute of Shanxi Agricultural University |
| IS 8544 | India | Sorghum Germplasm Resource Conservation Bank of Sorghum Research Institute of Shanxi Agricultural University |
| IS 22238 | India | Sorghum Germplasm Resource Conservation Bank of Sorghum Research Institute of Shanxi Agricultural University |
| ICS16B | India | Sorghum Germplasm Resource Conservation Bank of Sorghum Research Institute of Shanxi Agricultural University |
| S.O.102 | Japan | Sorghum Germplasm Resource Conservation Bank of Sorghum Research Institute of Shanxi Agricultural University |
| Fuji A | Japan | Sorghum Germplasm Resource Conservation Bank of Sorghum Research Institute of Shanxi Agricultural University |
| Qiye | China | Sorghum Germplasm Resource Conservation Bank of Sorghum Research Institute of Shanxi Agricultural University |
| IS 10477 | India | Sorghum Germplasm Resource Conservation Bank of Sorghum Research Institute of Shanxi Agricultural University |
| Jingyang Red Sorghum | China | Sorghum Germplasm Resource Conservation Bank of Sorghum Research Institute of Shanxi Agricultural University |
| Sixian Sugar Sorghum | China | Sorghum Germplasm Resource Conservation Bank of Sorghum Research Institute of Shanxi Agricultural University |
| PM 17413 | India | Sorghum Germplasm Resource Conservation Bank of Sorghum Research Institute of Shanxi Agricultural University |
| Huangluoshan | China | Sorghum Germplasm Resource Conservation Bank of Sorghum Research Institute of Shanxi Agricultural University |
| Funing Dwarf Sorghum | China | Sorghum Germplasm Resource Conservation Bank of Sorghum Research Institute of Shanxi Agricultural University |
| Daxing Sticky Sorghum | China | Sorghum Germplasm Resource Conservation Bank of Sorghum Research Institute of Shanxi Agricultural University |
| Bishon Kafir # 841 | U.S.A | Sorghum Germplasm Resource Conservation Bank of Sorghum Research Institute of Shanxi Agricultural University |
| Pedicel Half Bone | China | Sorghum Germplasm Resource Conservation Bank of Sorghum Research Institute of Shanxi Agricultural University |
| CHJ152 | China | Sorghum Germplasm Resource Conservation Bank of Sorghum Research Institute of Shanxi Agricultural University |
| IS 2279C | India | Sorghum Germplasm Resource Conservation Bank of Sorghum Research Institute of Shanxi Agricultural University |
| Hegari # 750 | U.S.A | Sorghum Germplasm Resource Conservation Bank of Sorghum Research Institute of Shanxi Agricultural University |
| ICSV 210 | India | Sorghum Germplasm Resource Conservation Bank of Sorghum Research Institute of Shanxi Agricultural University |
| CJ72 | China | Sorghum Germplasm Resource Conservation Bank of Sorghum Research Institute of Shanxi Agricultural University |
| IS 6440C | India | Sorghum Germplasm Resource Conservation Bank of Sorghum Research Institute of Shanxi Agricultural University |
| IS 955004 | India | Sorghum Germplasm Resource Conservation Bank of Sorghum Research Institute of Shanxi Agricultural University |
| 5-46796 | India | Sorghum Germplasm Resource Conservation Bank of Sorghum Research Institute of Shanxi Agricultural University |
| Tengchong Dwarf Sorghum | China | Sorghum Germplasm Resource Conservation Bank of Sorghum Research Institute of Shanxi Agricultural University |
| IS 339 | India | Sorghum Germplasm Resource Conservation Bank of Sorghum Research Institute of Shanxi Agricultural University |
| ICSR 41 | India | Sorghum Germplasm Resource Conservation Bank of Sorghum Research Institute of Shanxi Agricultural University |
| E35-1 | India | Sorghum Germplasm Resource Conservation Bank of Sorghum Research Institute of Shanxi Agricultural University |
| Chuotuihan | China | Sorghum Germplasm Resource Conservation Bank of Sorghum Research Institute of Shanxi Agricultural University |
| Bird Proof Kafer #662 | U.S.A | Sorghum Germplasm Resource Conservation Bank of Sorghum Research Institute of Shanxi Agricultural University |
| Geta Panicle | China | Sorghum Germplasm Resource Conservation Bank of Sorghum Research Institute of Shanxi Agricultural University |
| ICSV 197 | India | Sorghum Germplasm Resource Conservation Bank of Sorghum Research Institute of Shanxi Agricultural University |
| 77CS5388 | Mexico | Sorghum Germplasm Resource Conservation Bank of Sorghum Research Institute of Shanxi Agricultural University |
| Early Kalo Kansas | U.S.A | Sorghum Germplasm Resource Conservation Bank of Sorghum Research Institute of Shanxi Agricultural University |
| M1010B | China | Sorghum Germplasm Resource Conservation Bank of Sorghum Research Institute of Shanxi Agricultural University |
| HC356/5346 | China | Sorghum Germplasm Resource Conservation Bank of Sorghum Research Institute of Shanxi Agricultural University |
| Zhe43R | China | Sorghum Germplasm Resource Conservation Bank of Sorghum Research Institute of Shanxi Agricultural University |
| Y-121 | China | Sorghum Germplasm Resource Conservation Bank of Sorghum Research Institute of Shanxi Agricultural University |
| Xianmi Sorghum | China | Sorghum Germplasm Resource Conservation Bank of Sorghum Research Institute of Shanxi Agricultural University |
| 88BP28 | U.S.A | Sorghum Germplasm Resource Conservation Bank of Sorghum Research Institute of Shanxi Agricultural University |
| 6B | U.S.A | Sorghum Germplasm Resource Conservation Bank of Sorghum Research Institute of Shanxi Agricultural University |
| Purdu9-15-30 | U.S.A | Sorghum Germplasm Resource Conservation Bank of Sorghum Research Institute of Shanxi Agricultural University |
| P-10 | U.S.A | Sorghum Germplasm Resource Conservation Bank of Sorghum Research Institute of Shanxi Agricultural University |
| N91R | U.S.A | Sorghum Germplasm Resource Conservation Bank of Sorghum Research Institute of Shanxi Agricultural University |
| MalisorB4-7 | U.S.A | Sorghum Germplasm Resource Conservation Bank of Sorghum Research Institute of Shanxi Agricultural University |
| 94M4108 | U.S.A | Sorghum Germplasm Resource Conservation Bank of Sorghum Research Institute of Shanxi Agricultural University |
| 90CCEDN343 | U.S.A | Sorghum Germplasm Resource Conservation Bank of Sorghum Research Institute of Shanxi Agricultural University |
| Erchiban | China | Sorghum Germplasm Resource Conservation Bank of Sorghum Research Institute of Shanxi Agricultural University |
| Ke137R | China | Sorghum Germplasm Resource Conservation Bank of Sorghum Research Institute of Shanxi Agricultural University |
| Ke127R | China | Sorghum Germplasm Resource Conservation Bank of Sorghum Research Institute of Shanxi Agricultural University |
| Beiping No.7 | China | Sorghum Germplasm Resource Conservation Bank of Sorghum Research Institute of Shanxi Agricultural University |
| Beiping No.3 | China | Sorghum Germplasm Resource Conservation Bank of Sorghum Research Institute of Shanxi Agricultural University |
| Susong Sugar Sorghum | China | Sorghum Germplasm Resource Conservation Bank of Sorghum Research Institute of Shanxi Agricultural University |
| Fetarita # 182 | U.S.A | Sorghum Germplasm Resource Conservation Bank of Sorghum Research Institute of Shanxi Agricultural University |
| Fanchang Sugar Sorghum | China | Sorghum Germplasm Resource Conservation Bank of Sorghum Research Institute of Shanxi Agricultural University |
| CJJ164 | China | Sorghum Germplasm Resource Conservation Bank of Sorghum Research Institute of Shanxi Agricultural University |
| Haidian Red | China | Sorghum Germplasm Resource Conservation Bank of Sorghum Research Institute of Shanxi Agricultural University |
| Grohoma # 920 | U.S.A | Sorghum Germplasm Resource Conservation Bank of Sorghum Research Institute of Shanxi Agricultural University |
| Sweet Election 14(5006-3) | China | Sorghum Germplasm Resource Conservation Bank of Sorghum Research Institute of Shanxi Agricultural University |
| Guandong Sorghum | China | Sorghum Germplasm Resource Conservation Bank of Sorghum Research Institute of Shanxi Agricultural University |
| Zhenzhushan | China | Sorghum Germplasm Resource Conservation Bank of Sorghum Research Institute of Shanxi Agricultural University |
| Waibozhang | China | Sorghum Germplasm Resource Conservation Bank of Sorghum Research Institute of Shanxi Agricultural University |
| Habaifenzhi | China | Sorghum Germplasm Resource Conservation Bank of Sorghum Research Institute of Shanxi Agricultural University |
| CBJJ179 | China | Sorghum Germplasm Resource Conservation Bank of Sorghum Research Institute of Shanxi Agricultural University |
| Peitouhong | China | Sorghum Germplasm Resource Conservation Bank of Sorghum Research Institute of Shanxi Agricultural University |
| M-60839 | India | Sorghum Germplasm Resource Conservation Bank of Sorghum Research Institute of Shanxi Agricultural University |
| B8B | India | Sorghum Germplasm Resource Conservation Bank of Sorghum Research Institute of Shanxi Agricultural University |
| A16023-2 | India | Sorghum Germplasm Resource Conservation Bank of Sorghum Research Institute of Shanxi Agricultural University |
| L-402B | U.S.A | Sorghum Germplasm Resource Conservation Bank of Sorghum Research Institute of Shanxi Agricultural University |
| ICSB 102 | India | Sorghum Germplasm Resource Conservation Bank of Sorghum Research Institute of Shanxi Agricultural University |
| ICSB 88 | India | Sorghum Germplasm Resource Conservation Bank of Sorghum Research Institute of Shanxi Agricultural University |
| ICSB 70 | India | Sorghum Germplasm Resource Conservation Bank of Sorghum Research Institute of Shanxi Agricultural University |
| Lishihuang | China | Sorghum Germplasm Resource Conservation Bank of Sorghum Research Institute of Shanxi Agricultural University |
| IS 1134C | India | Sorghum Germplasm Resource Conservation Bank of Sorghum Research Institute of Shanxi Agricultural University |
| Changzi Red Hull Sorghum | China | Sorghum Germplasm Resource Conservation Bank of Sorghum Research Institute of Shanxi Agricultural University |
| ICSH 16 | India | Sorghum Germplasm Resource Conservation Bank of Sorghum Research Institute of Shanxi Agricultural University |
| ICSR88004 | India | Sorghum Germplasm Resource Conservation Bank of Sorghum Research Institute of Shanxi Agricultural University |
| ICSR 145 | India | Sorghum Germplasm Resource Conservation Bank of Sorghum Research Institute of Shanxi Agricultural University |
| ICSR 29 | India | Sorghum Germplasm Resource Conservation Bank of Sorghum Research Institute of Shanxi Agricultural University |
| GR108-90M24 | U.S.A | Sorghum Germplasm Resource Conservation Bank of Sorghum Research Institute of Shanxi Agricultural University |
| IS 1139C | India | Sorghum Germplasm Resource Conservation Bank of Sorghum Research Institute of Shanxi Agricultural University |
| Bwheelland-1 | U.S.A | Sorghum Germplasm Resource Conservation Bank of Sorghum Research Institute of Shanxi Agricultural University |
| Bwheelland-2 | U.S.A | Sorghum Germplasm Resource Conservation Bank of Sorghum Research Institute of Shanxi Agricultural University |
| 1131B | U.S.A | Sorghum Germplasm Resource Conservation Bank of Sorghum Research Institute of Shanxi Agricultural University |
| ICSV 202 | India | Sorghum Germplasm Resource Conservation Bank of Sorghum Research Institute of Shanxi Agricultural University |
| MR 933 | India | Sorghum Germplasm Resource Conservation Bank of Sorghum Research Institute of Shanxi Agricultural University |
| IS 7534C | India | Sorghum Germplasm Resource Conservation Bank of Sorghum Research Institute of Shanxi Agricultural University |
| IS 7506C | India | Sorghum Germplasm Resource Conservation Bank of Sorghum Research Institute of Shanxi Agricultural University |
| IS 7502C | India | Sorghum Germplasm Resource Conservation Bank of Sorghum Research Institute of Shanxi Agricultural University |
| PM 17681 | India | Sorghum Germplasm Resource Conservation Bank of Sorghum Research Institute of Shanxi Agricultural University |
| PM 12707 | India | Sorghum Germplasm Resource Conservation Bank of Sorghum Research Institute of Shanxi Agricultural University |
| PM 12653 | India | Sorghum Germplasm Resource Conservation Bank of Sorghum Research Institute of Shanxi Agricultural University |
| Broomcorn Sorghum | India | Sorghum Germplasm Resource Conservation Bank of Sorghum Research Institute of Shanxi Agricultural University |
| IS 18483 | India | Sorghum Germplasm Resource Conservation Bank of Sorghum Research Institute of Shanxi Agricultural University |
| 19504 | India | Sorghum Germplasm Resource Conservation Bank of Sorghum Research Institute of Shanxi Agricultural University |
| SPL 23R | India | Sorghum Germplasm Resource Conservation Bank of Sorghum Research Institute of Shanxi Agricultural University |
| PS 28020-2 | India | Sorghum Germplasm Resource Conservation Bank of Sorghum Research Institute of Shanxi Agricultural University |
| M 55799 | India | Sorghum Germplasm Resource Conservation Bank of Sorghum Research Institute of Shanxi Agricultural University |
| MB 926 | India | Sorghum Germplasm Resource Conservation Bank of Sorghum Research Institute of Shanxi Agricultural University |
| MB 906 | India | Sorghum Germplasm Resource Conservation Bank of Sorghum Research Institute of Shanxi Agricultural University |
| IS 24999 | India | Sorghum Germplasm Resource Conservation Bank of Sorghum Research Institute of Shanxi Agricultural University |
| 19518 | India | Sorghum Germplasm Resource Conservation Bank of Sorghum Research Institute of Shanxi Agricultural University |
| IS 1335C-1 | India | Sorghum Germplasm Resource Conservation Bank of Sorghum Research Institute of Shanxi Agricultural University |
| 5-46394 | India | Sorghum Germplasm Resource Conservation Bank of Sorghum Research Institute of Shanxi Agricultural University |
| Dorado | U.S.A | Sorghum Germplasm Resource Conservation Bank of Sorghum Research Institute of Shanxi Agricultural University |
| IS 16046 | India | Sorghum Germplasm Resource Conservation Bank of Sorghum Research Institute of Shanxi Agricultural University |
| IS 2177C | India | Sorghum Germplasm Resource Conservation Bank of Sorghum Research Institute of Shanxi Agricultural University |
| CJ311 | China | Sorghum Germplasm Resource Conservation Bank of Sorghum Research Institute of Shanxi Agricultural University |
| Nankou Sorghum | China | Sorghum Germplasm Resource Conservation Bank of Sorghum Research Institute of Shanxi Agricultural University |
| Baiwudalang | China | Sorghum Germplasm Resource Conservation Bank of Sorghum Research Institute of Shanxi Agricultural University |
| Baitaling White Sorghum | China | Sorghum Germplasm Resource Conservation Bank of Sorghum Research Institute of Shanxi Agricultural University |
| Heniyan | China | Sorghum Germplasm Resource Conservation Bank of Sorghum Research Institute of Shanxi Agricultural University |
| Leoti | U.S.A | Sorghum Germplasm Resource Conservation Bank of Sorghum Research Institute of Shanxi Agricultural University |
| IS 2508C | India | Sorghum Germplasm Resource Conservation Bank of Sorghum Research Institute of Shanxi Agricultural University |
| IS 3477C-2 | India | Sorghum Germplasm Resource Conservation Bank of Sorghum Research Institute of Shanxi Agricultural University |
| IS 3911C | India | Sorghum Germplasm Resource Conservation Bank of Sorghum Research Institute of Shanxi Agricultural University |
| IS 3071C | India | Sorghum Germplasm Resource Conservation Bank of Sorghum Research Institute of Shanxi Agricultural University |
| IS 4639C | India | Sorghum Germplasm Resource Conservation Bank of Sorghum Research Institute of Shanxi Agricultural University |
| IS 5554C | India | Sorghum Germplasm Resource Conservation Bank of Sorghum Research Institute of Shanxi Agricultural University |
| IS 5769C | India | Sorghum Germplasm Resource Conservation Bank of Sorghum Research Institute of Shanxi Agricultural University |
| IS 5892C | India | Sorghum Germplasm Resource Conservation Bank of Sorghum Research Institute of Shanxi Agricultural University |
| IS 8070 | India | Sorghum Germplasm Resource Conservation Bank of Sorghum Research Institute of Shanxi Agricultural University |
| IS 2662C | India | Sorghum Germplasm Resource Conservation Bank of Sorghum Research Institute of Shanxi Agricultural University |
| IS 6882C | India | Sorghum Germplasm Resource Conservation Bank of Sorghum Research Institute of Shanxi Agricultural University |
| IS 24989 | India | Sorghum Germplasm Resource Conservation Bank of Sorghum Research Institute of Shanxi Agricultural University |
| IS 7254C | India | Sorghum Germplasm Resource Conservation Bank of Sorghum Research Institute of Shanxi Agricultural University |
| Banas Durra | India | Sorghum Germplasm Resource Conservation Bank of Sorghum Research Institute of Shanxi Agricultural University |
| Atalas #899 | U.S.A | Sorghum Germplasm Resource Conservation Bank of Sorghum Research Institute of Shanxi Agricultural University |
| IS 2825 | India | Sorghum Germplasm Resource Conservation Bank of Sorghum Research Institute of Shanxi Agricultural University |
| ICSB 59 | India | Sorghum Germplasm Resource Conservation Bank of Sorghum Research Institute of Shanxi Agricultural University |
| ICSB 50 | India | Sorghum Germplasm Resource Conservation Bank of Sorghum Research Institute of Shanxi Agricultural University |
| Early Wheatland B | Japan | Sorghum Germplasm Resource Conservation Bank of Sorghum Research Institute of Shanxi Agricultural University |
| Early Hegarl | Japan | Sorghum Germplasm Resource Conservation Bank of Sorghum Research Institute of Shanxi Agricultural University |
| Early Kalo | Japan | Sorghum Germplasm Resource Conservation Bank of Sorghum Research Institute of Shanxi Agricultural University |
| Hegarl | Japan | Sorghum Germplasm Resource Conservation Bank of Sorghum Research Institute of Shanxi Agricultural University |
| Indian Amber | India | Sorghum Germplasm Resource Conservation Bank of Sorghum Research Institute of Shanxi Agricultural University |
| Dawn Kafir-1 | Africa | Sorghum Germplasm Resource Conservation Bank of Sorghum Research Institute of Shanxi Agricultural University |
| Dawn Kafir-2 | Africa | Sorghum Germplasm Resource Conservation Bank of Sorghum Research Institute of Shanxi Agricultural University |
| White Martin | Japan | Sorghum Germplasm Resource Conservation Bank of Sorghum Research Institute of Shanxi Agricultural University |
| Mocktak | Japan | Sorghum Germplasm Resource Conservation Bank of Sorghum Research Institute of Shanxi Agricultural University |
| Darso Ok #1 | U.S.A | Sorghum Germplasm Resource Conservation Bank of Sorghum Research Institute of Shanxi Agricultural University |
| Moctac Local | Japan | Sorghum Germplasm Resource Conservation Bank of Sorghum Research Institute of Shanxi Agricultural University |
| Suda ST11 | Japan | Sorghum Germplasm Resource Conservation Bank of Sorghum Research Institute of Shanxi Agricultural University |
| 14-T438-2 | Russia | Sorghum Germplasm Resource Conservation Bank of Sorghum Research Institute of Shanxi Agricultural University |
| 14-T456-1 | Russia | Sorghum Germplasm Resource Conservation Bank of Sorghum Research Institute of Shanxi Agricultural University |
| 14-T458-2 | Russia | Sorghum Germplasm Resource Conservation Bank of Sorghum Research Institute of Shanxi Agricultural University |
| 14-T529 | Russia | Sorghum Germplasm Resource Conservation Bank of Sorghum Research Institute of Shanxi Agricultural University |
| 14-T532 | Russia | Sorghum Germplasm Resource Conservation Bank of Sorghum Research Institute of Shanxi Agricultural University |
| 14-T545 | Russia | Sorghum Germplasm Resource Conservation Bank of Sorghum Research Institute of Shanxi Agricultural University |
| Sanchisan | China | Sorghum Germplasm Resource Conservation Bank of Sorghum Research Institute of Shanxi Agricultural University |
| Tx623B | U.S.A | Sorghum Germplasm Resource Conservation Bank of Sorghum Research Institute of Shanxi Agricultural University |
| V4B | China | Sorghum Germplasm Resource Conservation Bank of Sorghum Research Institute of Shanxi Agricultural University |
| Suxian Purple Hull | China | Sorghum Germplasm Resource Conservation Bank of Sorghum Research Institute of Shanxi Agricultural University |
| Taerhuang | China | Sorghum Germplasm Resource Conservation Bank of Sorghum Research Institute of Shanxi Agricultural University |
| Combine Kafir 60-2 | U.S.A | Sorghum Germplasm Resource Conservation Bank of Sorghum Research Institute of Shanxi Agricultural University |
| Shangqiu Black Hull-1 | China | Sorghum Germplasm Resource Conservation Bank of Sorghum Research Institute of Shanxi Agricultural University |
| Shangqiu Black Hull-2 | China | Sorghum Germplasm Resource Conservation Bank of Sorghum Research Institute of Shanxi Agricultural University |
| 741324 White | China | Sorghum Germplasm Resource Conservation Bank of Sorghum Research Institute of Shanxi Agricultural University |
| CSZ90 | China | Jiangsu Academy of Agricultural Sciences |
| CSZ59 | China | Jiangsu Academy of Agricultural Sciences |
| CSZ92 | China | Jiangsu Academy of Agricultural Sciences |
| CSZ64 | China | Jiangsu Academy of Agricultural Sciences |
| CSZ53 | China | Jiangsu Academy of Agricultural Sciences |
| CSZ66 | China | Jiangsu Academy of Agricultural Sciences |
| CSZ54 | China | Jiangsu Academy of Agricultural Sciences |
| CSZ41 | China | Jiangsu Academy of Agricultural Sciences |
| CSZ7 | China | Jiangsu Academy of Agricultural Sciences |
| CSZ57 | China | Jiangsu Academy of Agricultural Sciences |
| CSZ1 | China | Jiangsu Academy of Agricultural Sciences |
| CSZ13 | China | Jiangsu Academy of Agricultural Sciences |
| CSZ32 | China | Jiangsu Academy of Agricultural Sciences |
| CSZ19 | China | Jiangsu Academy of Agricultural Sciences |
| CSZ9 | China | Jiangsu Academy of Agricultural Sciences |
| CSZ60 | China | Jiangsu Academy of Agricultural Sciences |
| CSZ61 | China | Jiangsu Academy of Agricultural Sciences |
| CSz15 | China | Jiangsu Academy of Agricultural Sciences |
| CSZ3 | China | Jiangsu Academy of Agricultural Sciences |
| CSZ30 | China | Jiangsu Academy of Agricultural Sciences |
| CSZ31 | China | Jiangsu Academy of Agricultural Sciences |
| CSZ33 | China | Jiangsu Academy of Agricultural Sciences |
| CSZ36 | China | Jiangsu Academy of Agricultural Sciences |
| CSZ37 | China | Jiangsu Academy of Agricultural Sciences |
| CSZ39 | China | Jiangsu Academy of Agricultural Sciences |
| CSZ40 | China | Jiangsu Academy of Agricultural Sciences |
| CSZ58 | China | Jiangsu Academy of Agricultural Sciences |
| CSZ70 | China | Jiangsu Academy of Agricultural Sciences |

Table S2 The analysis of SNPs which exceeded threshold in all environments

| Trait | Env | Chr | Position | Pvalue | -log10P | Allele | MAF |
| --- | --- | --- | --- | --- | --- | --- | --- |
| TC | NJ2023 | Chr02 | 7982502 | 2.30E-08 | 7.64 | G/A | 0.075 |
| TC | NJ2023 | Chr02 | 7914181 | 5.49E-08 | 7.26 | C/T | 0.075 |
| TC | NJ2023 | Chr02 | 7914293 | 5.49E-08 | 7.26 | G/A | 0.075 |
| TC | NJ2023 | Chr02 | 7914444 | 5.49E-08 | 7.26 | A/G | 0.075 |
| TC | NJ2023 | Chr02 | 7916583 | 5.49E-08 | 7.26 | C/T | 0.075 |
| TC | NJ2023 | Chr02 | 7925267 | 5.49E-08 | 7.26 | A/G | 0.075 |
| TC | NJ2023 | Chr02 | 7925287 | 5.49E-08 | 7.26 | C/T | 0.075 |
| TC | NJ2023 | Chr02 | 7918330 | 5.72E-08 | 7.24 | C/T | 0.077 |
| TC | NJ2023 | Chr02 | 7918772 | 5.72E-08 | 7.24 | G/A | 0.077 |
| TC | NJ2023 | Chr02 | 7918571 | 5.79E-08 | 7.24 | G/A | 0.08 |
| TC | NJ2023 | Chr02 | 7959612 | 1.37E-07 | 6.86 | C/T | 0.075 |
| TC | NJ2023 | Chr02 | 7981695 | 1.56E-07 | 6.81 | C/G | 0.068 |
| TC | NJ2023 | Chr02 | 7963485 | 2.70E-07 | 6.57 | C/G | 0.077 |
| TC | NJ2023 | Chr02 | 7966252 | 2.70E-07 | 6.57 | A/G | 0.077 |
| TC | NJ2023 | Chr02 | 7958596 | 2.73E-07 | 6.56 | G/A | 0.08 |
| TC | NJ2023 | Chr02 | 7961191 | 2.83E-07 | 6.55 | C/T | 0.08 |
| TC | NJ2023 | Chr02 | 7966328 | 2.94E-07 | 6.53 | T/C | 0.082 |
| TC | NJ2023 | Chr02 | 7910597 | 7.35E-07 | 6.13 | C/T | 0.061 |
| TC | NJ2023 | Chr03 | 10645655 | 3.35E-07 | 6.47 | T/C | 0.075 |
| TC | NJ2023 | Chr03 | 10757809 | 2.82E-07 | 6.55 | A/T | 0.068 |
| TC | NJ2023 | Chr03 | 10843765 | 8.52E-08 | 7.07 | C/T | 0.068 |
| TC | NJ2023 | Chr03 | 10847028 | 4.12E-08 | 7.39 | C/T | 0.073 |
| TC | NJ2023 | Chr03 | 46660090 | 1.14E-07 | 6.94 | C/T | 0.07 |
| TC | NJ2024 | Chr02 | 7982502 | 5.17E-10 | 9.29 | G/A | 0.075 |
| TC | NJ2024 | Chr02 | 7914181 | 2.51E-09 | 8.60 | C/T | 0.075 |
| TC | NJ2024 | Chr02 | 7914293 | 2.51E-09 | 8.60 | G/A | 0.075 |
| TC | NJ2024 | Chr02 | 7914444 | 2.51E-09 | 8.60 | A/G | 0.075 |
| TC | NJ2024 | Chr02 | 7916583 | 2.51E-09 | 8.60 | C/T | 0.075 |
| TC | NJ2024 | Chr02 | 7925267 | 2.51E-09 | 8.60 | A/G | 0.075 |
| TC | NJ2024 | Chr02 | 7925287 | 2.51E-09 | 8.60 | C/T | 0.075 |
| TC | NJ2024 | Chr02 | 7918330 | 2.64E-09 | 8.58 | C/T | 0.077 |
| TC | NJ2024 | Chr02 | 7918772 | 2.64E-09 | 8.58 | G/A | 0.077 |
| TC | NJ2024 | Chr02 | 7918571 | 2.68E-09 | 8.57 | G/A | 0.08 |
| TC | NJ2024 | Chr02 | 7981695 | 3.96E-09 | 8.40 | C/G | 0.068 |
| TC | NJ2024 | Chr02 | 7959612 | 6.76E-09 | 8.17 | C/T | 0.075 |
| TC | NJ2024 | Chr02 | 7963485 | 8.90E-09 | 8.05 | C/G | 0.077 |
| TC | NJ2024 | Chr02 | 7966252 | 8.90E-09 | 8.05 | A/G | 0.077 |
| TC | NJ2024 | Chr02 | 7958596 | 8.96E-09 | 8.05 | G/A | 0.08 |
| TC | NJ2024 | Chr02 | 7961191 | 9.46E-09 | 8.02 | C/T | 0.08 |
| TC | NJ2024 | Chr02 | 7966328 | 9.54E-09 | 8.02 | T/C | 0.082 |
| TC | NJ2024 | Chr02 | 7910597 | 2.02E-07 | 6.69 | C/T | 0.061 |
| TC | NJ2024 | Chr02 | 7924188 | 4.22E-07 | 6.37 | C/T | 0.082 |
| TC | NJ2024 | Chr02 | 7599517 | 1.12E-06 | 5.95 | G/A | 0.07 |
| TC | NJ2024 | Chr03 | 25875862 | 8.56E-08 | 7.07 | A/C | 0.056 |
| TC | NJ2024 | Chr03 | 25876170 | 1.24E-06 | 5.91 | A/G | 0.061 |
| TC | YC2024 | Chr04 | 62062741 | 1.81E-06 | 5.74 | T/C | 0.07 |
| TC | YC2024 | Chr04 | 62081871 | 8.42E-07 | 6.07 | T/C | 0.056 |
| TC | YC2024 | Chr04 | 62083103 | 4.22E-07 | 6.37 | A/T | 0.059 |
| TC | YC2024 | Chr04 | 62083985 | 8.46E-07 | 6.07 | A/G | 0.059 |
| TC | YC2024 | Chr04 | 62089038 | 8.42E-07 | 6.07 | C/A | 0.056 |
| TC | YC2024 | Chr04 | 62094443 | 8.02E-07 | 6.10 | G/C | 0.059 |
| TC | YC2024 | Chr04 | 62096930 | 7.99E-07 | 6.10 | G/A | 0.059 |
| TC | YC2024 | Chr04 | 62098106 | 8.42E-07 | 6.07 | A/C | 0.056 |
| TC | YC2024 | Chr04 | 62099512 | 8.68E-07 | 6.06 | T/C | 0.061 |
| TC | YC2024 | Chr04 | 62099842 | 8.76E-07 | 6.06 | T/A | 0.059 |
| TC | YC2024 | Chr04 | 62104611 | 8.42E-07 | 6.07 | C/A | 0.056 |
| TC | YC2024 | Chr04 | 62115589 | 8.42E-07 | 6.07 | G/A | 0.056 |
| TC | YC2024 | Chr04 | 62119843 | 8.42E-07 | 6.07 | G/T | 0.056 |
| TC | YC2024 | Chr04 | 62122269 | 8.47E-07 | 6.07 | G/A | 0.059 |
| TC | YC2024 | Chr04 | 62208590 | 1.52E-06 | 5.82 | C/A | 0.056 |
| TC | YC2024 | Chr04 | 62208921 | 1.52E-06 | 5.82 | G/A | 0.056 |
| TC | YC2024 | Chr04 | 62209254 | 1.52E-06 | 5.82 | G/C | 0.056 |
| TC | YC2024 | Chr04 | 62209267 | 1.52E-06 | 5.82 | A/G | 0.056 |
| TC | YC2024 | Chr04 | 62209465 | 1.52E-06 | 5.82 | G/A | 0.056 |
| TC | YC2024 | Chr04 | 62209909 | 1.56E-06 | 5.81 | C/T | 0.059 |
| TC | YC2024 | Chr04 | 62210793 | 1.55E-06 | 5.81 | T/A | 0.059 |
| TC | YC2024 | Chr04 | 62214548 | 1.52E-06 | 5.82 | A/T | 0.056 |
| TC | BLUP | Chr02 | 5792036 | 1.59E-06 | 5.80 | G/A | 0.052 |
| TC | BLUP | Chr02 | 5854562 | 1.59E-06 | 5.80 | C/T | 0.052 |
| TC | BLUP | Chr02 | 7910597 | 2.01E-07 | 6.70 | C/T | 0.061 |
| TC | BLUP | Chr02 | 7914181 | 2.73E-09 | 8.56 | C/T | 0.075 |
| TC | BLUP | Chr02 | 7914293 | 2.73E-09 | 8.56 | G/A | 0.075 |
| TC | BLUP | Chr02 | 7914444 | 2.73E-09 | 8.56 | A/G | 0.075 |
| TC | BLUP | Chr02 | 7916583 | 2.73E-09 | 8.56 | C/T | 0.075 |
| TC | BLUP | Chr02 | 7918330 | 2.85E-09 | 8.55 | C/T | 0.077 |
| TC | BLUP | Chr02 | 7918571 | 2.90E-09 | 8.54 | G/A | 0.080 |
| TC | BLUP | Chr02 | 7918772 | 2.85E-09 | 8.55 | G/A | 0.077 |
| TC | BLUP | Chr02 | 7924188 | 3.47E-07 | 6.46 | C/T | 0.082 |
| TC | BLUP | Chr02 | 7925267 | 2.73E-09 | 8.56 | A/G | 0.075 |
| TC | BLUP | Chr02 | 7925287 | 2.73E-09 | 8.56 | C/T | 0.075 |
| TC | BLUP | Chr02 | 7958596 | 9.65E-09 | 8.02 | G/A | 0.080 |
| TC | BLUP | Chr02 | 7959612 | 4.30E-09 | 8.37 | C/T | 0.075 |
| TC | BLUP | Chr02 | 7961191 | 1.01E-08 | 8.00 | C/T | 0.080 |
| TC | BLUP | Chr02 | 7963485 | 9.67E-09 | 8.01 | C/G | 0.077 |
| TC | BLUP | Chr02 | 7966252 | 9.67E-09 | 8.01 | A/G | 0.077 |
| TC | BLUP | Chr02 | 7966328 | 1.06E-08 | 7.97 | T/C | 0.082 |
| TC | BLUP | Chr02 | 7981695 | 7.26E-09 | 8.14 | C/G | 0.068 |
| TC | BLUP | Chr02 | 7982502 | 1.02E-09 | 8.99 | G/A | 0.075 |
| TC | BLUP | Chr03 | 10847028 | 7.89E-07 | 6.10 | C/T | 0.073 |
| TC | BLUP | Chr03 | 10843765 | 1.66E-06 | 5.78 | C/T | 0.068 |
| SC | NJ2023 | Chr02 | 45158091 | 3.82E-07 | 6.42 | G/A | 0.066 |
| SC | NJ2023 | Chr02 | 45158616 | 1.72E-06 | 5.76 | T/C | 0.063 |
| SC | NJ2023 | Chr02 | 45158626 | 1.72E-06 | 5.76 | C/T | 0.063 |
| SC | NJ2023 | Chr04 | 68013997 | 1.99E-07 | 6.70 | G/A | 0.12 |
| SC | NJ2023 | Chr04 | 68014104 | 2.07E-07 | 6.68 | G/A | 0.129 |
| SC | NJ2023 | Chr04 | 68014123 | 1.90E-07 | 6.72 | C/T | 0.117 |
| SC | NJ2023 | Chr04 | 68014140 | 2.07E-07 | 6.68 | C/T | 0.129 |
| SC | NJ2023 | Chr04 | 68014199 | 2.21E-07 | 6.66 | T/C | 0.131 |
| SC | NJ2023 | Chr04 | 68014212 | 1.99E-07 | 6.70 | A/C | 0.122 |
| SC | NJ2023 | Chr04 | 68014364 | 2.07E-07 | 6.68 | G/T | 0.129 |
| SC | NJ2023 | Chr04 | 68014499 | 7.89E-07 | 6.10 | T/G | 0.134 |
| SC | NJ2023 | Chr04 | 68014517 | 2.21E-07 | 6.66 | T/C | 0.131 |
| SC | NJ2023 | Chr04 | 68014542 | 1.89E-07 | 6.72 | C/T | 0.122 |
| SC | NJ2023 | Chr04 | 68014619 | 2.58E-07 | 6.59 | G/A | 0.134 |
| SC | NJ2023 | Chr04 | 68014832 | 2.21E-07 | 6.66 | A/G | 0.131 |
| SC | NJ2023 | Chr04 | 68014847 | 2.21E-07 | 6.66 | C/T | 0.136 |
| SC | NJ2023 | Chr04 | 68014973 | 2.21E-07 | 6.66 | A/T | 0.131 |
| SC | NJ2023 | Chr04 | 68014985 | 2.21E-07 | 6.66 | T/A | 0.131 |
| SC | NJ2023 | Chr04 | 68015179 | 8.17E-07 | 6.09 | C/T | 0.122 |
| SC | NJ2023 | Chr04 | 68015294 | 7.89E-07 | 6.10 | G/T | 0.134 |
| SC | NJ2023 | Chr04 | 68015309 | 7.89E-07 | 6.10 | C/T | 0.134 |
| SC | NJ2023 | Chr04 | 68015474 | 2.21E-07 | 6.66 | G/A | 0.131 |
| SC | NJ2023 | Chr04 | 68015489 | 2.21E-07 | 6.66 | T/C | 0.131 |
| SC | NJ2023 | Chr04 | 68015513 | 2.07E-07 | 6.68 | A/G | 0.129 |
| SC | NJ2023 | Chr04 | 68015528 | 2.07E-07 | 6.68 | T/C | 0.129 |
| SC | NJ2023 | Chr04 | 68015648 | 2.21E-07 | 6.66 | C/T | 0.131 |
| SC | NJ2023 | Chr04 | 68015696 | 1.54E-07 | 6.81 | A/T | 0.134 |
| SC | NJ2023 | Chr04 | 68015720 | 1.54E-07 | 6.81 | A/G | 0.134 |
| SC | NJ2023 | Chr04 | 68015780 | 2.21E-07 | 6.66 | C/T | 0.131 |
| SC | NJ2023 | Chr04 | 68015795 | 2.21E-07 | 6.66 | T/G | 0.131 |
| SC | NJ2023 | Chr04 | 68015912 | 2.07E-07 | 6.68 | A/C | 0.129 |
| SC | NJ2023 | Chr04 | 68016029 | 5.88E-08 | 7.23 | C/T | 0.129 |
| SC | NJ2023 | Chr04 | 68016048 | 5.88E-08 | 7.23 | G/T | 0.129 |
| SC | NJ2023 | Chr04 | 68016058 | 7.42E-07 | 6.13 | C/A | 0.131 |
| SC | NJ2023 | Chr04 | 68016094 | 9.75E-07 | 6.01 | C/G | 0.117 |
| SC | NJ2023 | Chr04 | 68016638 | 7.89E-07 | 6.10 | A/G | 0.134 |
| SC | NJ2023 | Chr04 | 68016734 | 7.89E-07 | 6.10 | A/G | 0.134 |
| SC | NJ2023 | Chr04 | 68016977 | 6.93E-08 | 7.16 | G/C | 0.131 |
| SC | NJ2023 | Chr04 | 68016992 | 6.57E-08 | 7.18 | G/T | 0.129 |
| SC | NJ2023 | Chr04 | 68017001 | 6.57E-08 | 7.18 | T/C | 0.129 |
| SC | NJ2023 | Chr04 | 68017007 | 6.57E-08 | 7.18 | T/C | 0.129 |
| SC | NJ2023 | Chr04 | 68017040 | 2.07E-07 | 6.68 | A/G | 0.129 |
| SC | NJ2023 | Chr04 | 68017111 | 2.21E-07 | 6.66 | G/A | 0.131 |
| SC | NJ2023 | Chr04 | 68017142 | 1.90E-07 | 6.72 | C/T | 0.129 |
| SC | NJ2023 | Chr04 | 68017163 | 1.90E-07 | 6.72 | A/T | 0.129 |
| SC | NJ2023 | Chr04 | 68017178 | 1.91E-07 | 6.72 | A/T | 0.131 |
| SC | NJ2023 | Chr04 | 68017318 | 3.81E-07 | 6.42 | G/A | 0.12 |
| SC | NJ2023 | Chr04 | 68017388 | 2.14E-07 | 6.67 | A/G | 0.134 |
| SC | NJ2023 | Chr04 | 68017706 | 2.21E-07 | 6.66 | G/A | 0.131 |
| SC | NJ2023 | Chr04 | 68017733 | 1.86E-07 | 6.73 | C/T | 0.122 |
| SC | NJ2023 | Chr04 | 68017772 | 2.21E-07 | 6.66 | C/A | 0.131 |
| SC | NJ2023 | Chr04 | 68017876 | 2.96E-07 | 6.53 | G/A | 0.115 |
| SC | NJ2023 | Chr04 | 68017891 | 2.07E-07 | 6.68 | C/T | 0.129 |
| SC | NJ2023 | Chr04 | 68018264 | 2.21E-07 | 6.66 | G/A | 0.131 |
| SC | NJ2023 | Chr04 | 68018865 | 2.39E-07 | 6.62 | G/A | 0.134 |
| SC | NJ2023 | Chr04 | 68018940 | 2.53E-07 | 6.60 | A/C | 0.124 |
| SC | NJ2023 | Chr04 | 68112330 | 3.82E-07 | 6.42 | A/G | 0.066 |
| SC | NJ2024 | Chr02 | 45158091 | 5.16E-07 | 6.29 | G/A | 0.066 |
| SC | NJ2024 | Chr02 | 45158616 | 1.60E-06 | 5.80 | T/C | 0.063 |
| SC | NJ2024 | Chr02 | 45158626 | 1.60E-06 | 5.80 | C/T | 0.063 |
| SC | NJ2024 | Chr04 | 12113446 | 1.72E-06 | 5.76 | G/C | 0.075 |
| SC | NJ2024 | Chr04 | 12115042 | 1.67E-06 | 5.78 | C/G | 0.075 |
| SC | NJ2024 | Chr04 | 12115123 | 1.51E-06 | 5.82 | A/G | 0.077 |
| SC | NJ2024 | Chr04 | 12115306 | 1.65E-06 | 5.78 | C/A | 0.075 |
| SC | NJ2024 | Chr04 | 12134206 | 1.75E-06 | 5.76 | G/A | 0.073 |
| SC | NJ2024 | Chr04 | 12134212 | 1.75E-06 | 5.76 | C/T | 0.075 |
| SC | NJ2024 | Chr04 | 12139848 | 1.48E-06 | 5.83 | C/G | 0.077 |
| SC | NJ2024 | Chr04 | 12139856 | 1.48E-06 | 5.83 | T/G | 0.077 |
| SC | NJ2024 | Chr04 | 12142267 | 1.76E-06 | 5.75 | C/T | 0.077 |
| SC | NJ2024 | Chr04 | 12142311 | 1.75E-06 | 5.76 | G/A | 0.075 |
| SC | NJ2024 | Chr04 | 12143659 | 1.67E-06 | 5.78 | G/A | 0.077 |
| SC | NJ2024 | Chr04 | 12163865 | 4.44E-08 | 7.35 | G/A | 0.052 |
| SC | NJ2024 | Chr04 | 12168371 | 4.44E-08 | 7.35 | C/T | 0.052 |
| SC | NJ2024 | Chr04 | 12170710 | 4.44E-08 | 7.35 | C/T | 0.052 |
| SC | NJ2024 | Chr04 | 12177838 | 5.22E-07 | 6.28 | C/T | 0.077 |
| SC | NJ2024 | Chr04 | 68112330 | 5.16E-07 | 6.29 | A/G | 0.066 |
| SC | YC2024 | Chr01 | 22782482 | 1.49E-06 | 5.83 | C/T | 0.244 |
| SC | YC2024 | Chr01 | 22782600 | 4.21E-07 | 6.38 | G/A | 0.239 |
| SC | YC2024 | Chr01 | 22783048 | 1.41E-06 | 5.85 | G/A | 0.232 |
| SC | YC2024 | Chr01 | 22785452 | 9.42E-07 | 6.03 | C/T | 0.246 |
| SC | YC2024 | Chr01 | 22785480 | 7.56E-07 | 6.12 | G/A | 0.242 |
| SC | YC2024 | Chr01 | 22785539 | 4.36E-07 | 6.36 | G/A | 0.239 |
| SC | YC2024 | Chr01 | 22785551 | 7.35E-07 | 6.13 | G/A | 0.239 |
| SC | YC2024 | Chr01 | 35968444 | 1.50E-06 | 5.82 | T/A | 0.244 |
| SC | YC2024 | Chr01 | 35968459 | 1.50E-06 | 5.82 | T/C | 0.244 |
| SC | YC2024 | Chr02 | 47725537 | 1.12E-06 | 5.95 | T/C | 0.241 |
| SC | YC2024 | Chr02 | 47725687 | 1.49E-06 | 5.83 | C/T | 0.244 |
| SC | BLUP | Chr04 | 12106022 | 1.66E-06 | 5.78 | A/G | 0.073 |
| SC | BLUP | Chr04 | 12109930 | 1.72E-06 | 5.76 | T/C | 0.070 |
| SC | BLUP | Chr04 | 12109969 | 1.67E-06 | 5.78 | G/A | 0.073 |
| SC | BLUP | Chr04 | 12110445 | 1.67E-06 | 5.78 | T/C | 0.073 |
| SC | BLUP | Chr04 | 12110544 | 1.67E-06 | 5.78 | C/G | 0.073 |
| SC | BLUP | Chr04 | 12111287 | 1.67E-06 | 5.78 | C/T | 0.073 |
| SC | BLUP | Chr04 | 12111399 | 1.67E-06 | 5.78 | G/A | 0.073 |
| SC | BLUP | Chr04 | 12111545 | 1.67E-06 | 5.78 | G/T | 0.073 |
| SC | BLUP | Chr04 | 12111558 | 1.67E-06 | 5.78 | C/T | 0.073 |
| SC | BLUP | Chr04 | 12111639 | 1.67E-06 | 5.78 | C/T | 0.073 |
| SC | BLUP | Chr04 | 12111711 | 1.67E-06 | 5.78 | C/T | 0.073 |
| SC | BLUP | Chr04 | 12111719 | 1.67E-06 | 5.78 | T/C | 0.073 |
| SC | BLUP | Chr04 | 12111738 | 1.67E-06 | 5.78 | T/C | 0.073 |
| SC | BLUP | Chr04 | 12111866 | 1.67E-06 | 5.78 | A/G | 0.073 |
| SC | BLUP | Chr04 | 12112069 | 1.59E-06 | 5.80 | G/A | 0.075 |
| SC | BLUP | Chr04 | 12112077 | 1.59E-06 | 5.80 | T/C | 0.075 |
| SC | BLUP | Chr04 | 12112174 | 1.67E-06 | 5.78 | C/T | 0.073 |
| SC | BLUP | Chr04 | 12112335 | 1.67E-06 | 5.78 | G/A | 0.075 |
| SC | BLUP | Chr04 | 12112423 | 1.67E-06 | 5.78 | G/A | 0.073 |
| SC | BLUP | Chr04 | 12112449 | 1.67E-06 | 5.78 | A/G | 0.073 |
| SC | BLUP | Chr04 | 12112473 | 1.67E-06 | 5.78 | T/C | 0.073 |
| SC | BLUP | Chr04 | 12112516 | 1.67E-06 | 5.78 | A/C | 0.075 |
| SC | BLUP | Chr04 | 12112522 | 1.67E-06 | 5.78 | A/G | 0.075 |
| SC | BLUP | Chr04 | 12112540 | 1.67E-06 | 5.78 | G/A | 0.073 |
| SC | BLUP | Chr04 | 12112566 | 1.67E-06 | 5.78 | C/T | 0.073 |
| SC | BLUP | Chr04 | 12112673 | 1.67E-06 | 5.78 | G/T | 0.073 |
| SC | BLUP | Chr04 | 12112716 | 1.67E-06 | 5.78 | T/C | 0.075 |
| SC | BLUP | Chr04 | 12112890 | 1.67E-06 | 5.78 | G/T | 0.073 |
| SC | BLUP | Chr04 | 12113024 | 1.67E-06 | 5.78 | G/C | 0.073 |
| SC | BLUP | Chr04 | 12113108 | 1.67E-06 | 5.78 | A/G | 0.073 |
| SC | BLUP | Chr04 | 12113285 | 1.67E-06 | 5.78 | C/T | 0.073 |
| SC | BLUP | Chr04 | 12113294 | 1.67E-06 | 5.78 | G/A | 0.073 |
| SC | BLUP | Chr04 | 12113446 | 1.62E-06 | 5.79 | G/C | 0.075 |
| SC | BLUP | Chr04 | 12113507 | 1.81E-06 | 5.74 | T/C | 0.075 |
| SC | BLUP | Chr04 | 12113569 | 1.67E-06 | 5.78 | T/C | 0.073 |
| SC | BLUP | Chr04 | 12113623 | 1.71E-06 | 5.77 | A/T | 0.077 |
| SC | BLUP | Chr04 | 12113633 | 1.71E-06 | 5.77 | G/T | 0.077 |
| SC | BLUP | Chr04 | 12113822 | 1.67E-06 | 5.78 | A/T | 0.073 |
| SC | BLUP | Chr04 | 12114115 | 1.67E-06 | 5.78 | C/A | 0.073 |
| SC | BLUP | Chr04 | 12114162 | 1.67E-06 | 5.78 | T/C | 0.073 |
| SC | BLUP | Chr04 | 12114206 | 1.67E-06 | 5.78 | T/C | 0.073 |
| SC | BLUP | Chr04 | 12114263 | 1.67E-06 | 5.78 | C/T | 0.073 |
| SC | BLUP | Chr04 | 12114282 | 1.67E-06 | 5.78 | T/C | 0.073 |
| SC | BLUP | Chr04 | 12114289 | 1.67E-06 | 5.78 | G/T | 0.073 |
| SC | BLUP | Chr04 | 12114328 | 1.67E-06 | 5.78 | A/T | 0.073 |
| SC | BLUP | Chr04 | 12114433 | 1.67E-06 | 5.78 | C/T | 0.073 |
| SC | BLUP | Chr04 | 12114458 | 1.67E-06 | 5.78 | A/C | 0.073 |
| SC | BLUP | Chr04 | 12114596 | 1.65E-06 | 5.78 | C/G | 0.075 |
| SC | BLUP | Chr04 | 12114717 | 1.67E-06 | 5.78 | A/G | 0.073 |
| SC | BLUP | Chr04 | 12114786 | 1.67E-06 | 5.78 | A/G | 0.073 |
| SC | BLUP | Chr04 | 12114862 | 1.67E-06 | 5.78 | G/C | 0.073 |
| SC | BLUP | Chr04 | 12114869 | 1.67E-06 | 5.78 | T/C | 0.073 |
| SC | BLUP | Chr04 | 12115008 | 1.67E-06 | 5.78 | G/A | 0.073 |
| SC | BLUP | Chr04 | 12115017 | 1.67E-06 | 5.78 | T/G | 0.073 |
| SC | BLUP | Chr04 | 12115042 | 1.62E-06 | 5.79 | C/G | 0.075 |
| SC | BLUP | Chr04 | 12115089 | 1.67E-06 | 5.78 | A/G | 0.073 |
| SC | BLUP | Chr04 | 12115123 | 1.34E-06 | 5.87 | A/G | 0.077 |
| SC | BLUP | Chr04 | 12115149 | 1.67E-06 | 5.78 | T/C | 0.073 |
| SC | BLUP | Chr04 | 12115238 | 1.67E-06 | 5.78 | T/C | 0.073 |
| SC | BLUP | Chr04 | 12115306 | 1.42E-06 | 5.85 | C/A | 0.075 |
| SC | BLUP | Chr04 | 12115326 | 1.67E-06 | 5.78 | T/C | 0.073 |
| SC | BLUP | Chr04 | 12115341 | 1.67E-06 | 5.78 | G/A | 0.073 |
| SC | BLUP | Chr04 | 12115443 | 1.67E-06 | 5.78 | T/G | 0.073 |
| SC | BLUP | Chr04 | 12115452 | 1.67E-06 | 5.78 | C/A | 0.073 |
| SC | BLUP | Chr04 | 12115483 | 1.72E-06 | 5.76 | A/T | 0.075 |
| SC | BLUP | Chr04 | 12115531 | 1.67E-06 | 5.78 | T/C | 0.073 |
| SC | BLUP | Chr04 | 12115574 | 1.67E-06 | 5.78 | T/G | 0.073 |
| SC | BLUP | Chr04 | 12115595 | 1.67E-06 | 5.78 | T/G | 0.073 |
| SC | BLUP | Chr04 | 12115679 | 1.67E-06 | 5.78 | A/G | 0.073 |
| SC | BLUP | Chr04 | 12115773 | 1.67E-06 | 5.78 | C/G | 0.073 |
| SC | BLUP | Chr04 | 12115820 | 1.67E-06 | 5.78 | G/C | 0.073 |
| SC | BLUP | Chr04 | 12115863 | 1.67E-06 | 5.78 | C/T | 0.073 |
| SC | BLUP | Chr04 | 12116161 | 1.67E-06 | 5.78 | T/A | 0.073 |
| SC | BLUP | Chr04 | 12116226 | 1.67E-06 | 5.78 | C/A | 0.073 |
| SC | BLUP | Chr04 | 12116329 | 1.67E-06 | 5.78 | C/A | 0.073 |
| SC | BLUP | Chr04 | 12116337 | 1.67E-06 | 5.78 | G/A | 0.073 |
| SC | BLUP | Chr04 | 12116455 | 1.67E-06 | 5.78 | G/T | 0.073 |
| SC | BLUP | Chr04 | 12116550 | 1.67E-06 | 5.78 | C/T | 0.073 |
| SC | BLUP | Chr04 | 12116559 | 1.67E-06 | 5.78 | A/C | 0.073 |
| SC | BLUP | Chr04 | 12116568 | 1.67E-06 | 5.78 | G/A | 0.073 |
| SC | BLUP | Chr04 | 12116583 | 1.67E-06 | 5.78 | C/T | 0.073 |
| SC | BLUP | Chr04 | 12116636 | 1.67E-06 | 5.78 | C/A | 0.073 |
| SC | BLUP | Chr04 | 12116697 | 1.71E-06 | 5.77 | T/C | 0.077 |
| SC | BLUP | Chr04 | 12116752 | 1.67E-06 | 5.78 | T/C | 0.073 |
| SC | BLUP | Chr04 | 12116781 | 1.67E-06 | 5.78 | A/G | 0.073 |
| SC | BLUP | Chr04 | 12116793 | 1.67E-06 | 5.78 | C/G | 0.073 |
| SC | BLUP | Chr04 | 12116823 | 1.67E-06 | 5.78 | C/T | 0.073 |
| SC | BLUP | Chr04 | 12116873 | 1.67E-06 | 5.78 | G/A | 0.073 |
| SC | BLUP | Chr04 | 12116896 | 1.67E-06 | 5.78 | T/C | 0.073 |
| SC | BLUP | Chr04 | 12117166 | 1.67E-06 | 5.78 | A/C | 0.073 |
| SC | BLUP | Chr04 | 12117219 | 1.67E-06 | 5.78 | C/T | 0.073 |
| SC | BLUP | Chr04 | 12117257 | 1.67E-06 | 5.78 | T/C | 0.073 |
| SC | BLUP | Chr04 | 12117675 | 1.67E-06 | 5.78 | T/C | 0.075 |
| SC | BLUP | Chr04 | 12117731 | 1.67E-06 | 5.78 | C/T | 0.075 |
| SC | BLUP | Chr04 | 12117767 | 1.67E-06 | 5.78 | C/T | 0.073 |
| SC | BLUP | Chr04 | 12117956 | 1.67E-06 | 5.78 | T/C | 0.073 |
| SC | BLUP | Chr04 | 12118005 | 1.67E-06 | 5.78 | T/C | 0.073 |
| SC | BLUP | Chr04 | 12118102 | 1.67E-06 | 5.78 | C/G | 0.073 |
| SC | BLUP | Chr04 | 12118118 | 1.67E-06 | 5.78 | C/G | 0.073 |
| SC | BLUP | Chr04 | 12118284 | 1.67E-06 | 5.78 | T/C | 0.073 |
| SC | BLUP | Chr04 | 12118321 | 1.67E-06 | 5.78 | C/T | 0.075 |
| SC | BLUP | Chr04 | 12118352 | 1.67E-06 | 5.78 | A/G | 0.073 |
| SC | BLUP | Chr04 | 12118464 | 1.67E-06 | 5.78 | T/G | 0.073 |
| SC | BLUP | Chr04 | 12118498 | 1.67E-06 | 5.78 | C/A | 0.075 |
| SC | BLUP | Chr04 | 12118573 | 1.67E-06 | 5.78 | G/A | 0.073 |
| SC | BLUP | Chr04 | 12118643 | 1.67E-06 | 5.78 | A/T | 0.073 |
| SC | BLUP | Chr04 | 12118652 | 1.67E-06 | 5.78 | A/G | 0.073 |
| SC | BLUP | Chr04 | 12120437 | 1.73E-06 | 5.76 | C/G | 0.075 |
| SC | BLUP | Chr04 | 12120443 | 1.73E-06 | 5.76 | G/C | 0.075 |
| SC | BLUP | Chr04 | 12120467 | 1.67E-06 | 5.78 | A/C | 0.073 |
| SC | BLUP | Chr04 | 12120564 | 1.67E-06 | 5.78 | A/G | 0.073 |
| SC | BLUP | Chr04 | 12120587 | 1.67E-06 | 5.78 | A/G | 0.073 |
| SC | BLUP | Chr04 | 12120606 | 1.67E-06 | 5.78 | A/G | 0.073 |
| SC | BLUP | Chr04 | 12120764 | 1.67E-06 | 5.78 | C/G | 0.073 |
| SC | BLUP | Chr04 | 12120816 | 1.67E-06 | 5.78 | A/G | 0.073 |
| SC | BLUP | Chr04 | 12120873 | 1.67E-06 | 5.78 | T/G | 0.073 |
| SC | BLUP | Chr04 | 12120914 | 1.67E-06 | 5.78 | G/A | 0.073 |
| SC | BLUP | Chr04 | 12121001 | 1.70E-06 | 5.77 | C/T | 0.077 |
| SC | BLUP | Chr04 | 12121112 | 1.67E-06 | 5.78 | A/G | 0.073 |
| SC | BLUP | Chr04 | 12121128 | 1.67E-06 | 5.78 | C/T | 0.073 |
| SC | BLUP | Chr04 | 12121238 | 1.68E-06 | 5.77 | C/A | 0.077 |
| SC | BLUP | Chr04 | 12121320 | 1.67E-06 | 5.78 | G/T | 0.073 |
| SC | BLUP | Chr04 | 12121335 | 1.67E-06 | 5.78 | C/T | 0.073 |
| SC | BLUP | Chr04 | 12121374 | 1.67E-06 | 5.78 | G/A | 0.073 |
| SC | BLUP | Chr04 | 12121448 | 1.67E-06 | 5.78 | A/G | 0.075 |
| SC | BLUP | Chr04 | 12134206 | 1.61E-06 | 5.79 | G/A | 0.073 |
| SC | BLUP | Chr04 | 12134212 | 1.61E-06 | 5.79 | C/T | 0.075 |
| SC | BLUP | Chr04 | 12138644 | 1.67E-06 | 5.78 | G/T | 0.073 |
| SC | BLUP | Chr04 | 12138683 | 1.67E-06 | 5.78 | A/G | 0.073 |
| SC | BLUP | Chr04 | 12138700 | 1.67E-06 | 5.78 | T/A | 0.073 |
| SC | BLUP | Chr04 | 12138751 | 1.67E-06 | 5.78 | A/G | 0.073 |
| SC | BLUP | Chr04 | 12139587 | 1.67E-06 | 5.78 | A/G | 0.073 |
| SC | BLUP | Chr04 | 12139640 | 1.72E-06 | 5.76 | G/A | 0.075 |
| SC | BLUP | Chr04 | 12139848 | 1.28E-06 | 5.89 | C/G | 0.077 |
| SC | BLUP | Chr04 | 12139856 | 1.28E-06 | 5.89 | T/G | 0.077 |
| SC | BLUP | Chr04 | 12139983 | 1.67E-06 | 5.78 | A/T | 0.073 |
| SC | BLUP | Chr04 | 12140098 | 1.67E-06 | 5.78 | C/T | 0.073 |
| SC | BLUP | Chr04 | 12140149 | 1.68E-06 | 5.77 | G/T | 0.075 |
| SC | BLUP | Chr04 | 12140164 | 1.67E-06 | 5.78 | G/A | 0.073 |
| SC | BLUP | Chr04 | 12142267 | 1.51E-06 | 5.82 | C/T | 0.077 |
| SC | BLUP | Chr04 | 12142311 | 1.51E-06 | 5.82 | G/A | 0.075 |
| SC | BLUP | Chr04 | 12142369 | 1.66E-06 | 5.78 | G/A | 0.075 |
| SC | BLUP | Chr04 | 12142404 | 1.67E-06 | 5.78 | C/T | 0.073 |
| SC | BLUP | Chr04 | 12142441 | 1.67E-06 | 5.78 | A/G | 0.073 |
| SC | BLUP | Chr04 | 12142495 | 1.67E-06 | 5.78 | A/G | 0.073 |
| SC | BLUP | Chr04 | 12142509 | 1.67E-06 | 5.78 | T/C | 0.073 |
| SC | BLUP | Chr04 | 12142549 | 1.67E-06 | 5.78 | G/A | 0.073 |
| SC | BLUP | Chr04 | 12143468 | 1.67E-06 | 5.78 | C/T | 0.073 |
| SC | BLUP | Chr04 | 12143533 | 1.67E-06 | 5.78 | T/G | 0.073 |
| SC | BLUP | Chr04 | 12143540 | 1.67E-06 | 5.78 | G/A | 0.073 |
| SC | BLUP | Chr04 | 12143569 | 1.67E-06 | 5.78 | A/G | 0.073 |
| SC | BLUP | Chr04 | 12143642 | 1.67E-06 | 5.78 | T/C | 0.073 |
| SC | BLUP | Chr04 | 12143659 | 1.60E-06 | 5.80 | C/T | 0.077 |
| SC | BLUP | Chr04 | 12143680 | 1.71E-06 | 5.77 | T/A | 0.075 |
| SC | BLUP | Chr04 | 12143772 | 1.75E-06 | 5.76 | G/A | 0.068 |
| SC | BLUP | Chr04 | 12143795 | 1.75E-06 | 5.76 | T/C | 0.068 |
| SC | BLUP | Chr04 | 12163865 | 6.14E-07 | 6.21 | G/A | 0.052 |
| SC | BLUP | Chr04 | 12168371 | 6.14E-07 | 6.21 | C/T | 0.052 |
| SC | BLUP | Chr04 | 12170710 | 6.14E-07 | 6.21 | C/T | 0.052 |
| SC | BLUP | Chr04 | 12177838 | 1.06E-06 | 5.97 | C/T | 0.077 |
| SC | BLUP | Chr04 | 68014847 | 2.67E-07 | 6.57 | C/T | 0.136 |

Env: environment; Chr: chromosome; MAF: minor allele frequency; TC: tannin content; SC: starch content

Table S3 The variations of the haplotypes for the sorghum Sobic.002G076600

| Alleles | T/C | T/G |
| --- | --- | --- |
| Chromosome | Chr02 | Chr02 |
| Position | 7979912 | 7983795 |
| Hap1 | T | G |
| Hap2 | C | T |
| Hap3 | T | T |

Table S4 The variations of the haplotypes for the sorghum Sobic.003G118266

| Alleles | C/T | C/G | G/A | G/A | G/A | A/G | C/T |
| --- | --- | --- | --- | --- | --- | --- | --- |
| Chromosome | Chr03 | Chr03 | Chr03 | Chr03 | Chr03 | Chr03 | Chr03 |
| Position | 10693515 | 10693588 | 10693721 | 10693743 | 10693983 | 10694005 | 10694089 |
| Hap1 | C | C | A | A | A | A | C |
| Hap2 | C | C | G | G | G | A | C |
| Hap3 | C | C | G | G | G | A | T |
| Hap4 | C | C | G | G | G | G | C |
| Hap5 | T | G | G | G | G | G | C |

Table S5 The variations of the haplotypes for the sorghum Sobic.004G353100

| Alleles | A/G | G/T | A/G | C/T | G/A | A/G | G/A | C/T | G/T |
| --- | --- | --- | --- | --- | --- | --- | --- | --- | --- |
| Chromosome | Chr04 | Chr04 | Chr04 | Chr04 | Chr04 | Chr04 | Chr04 | Chr04 | Chr04 |
| Position | 68133678 | 68135000 | 68135429 | 68135516 | 68135592 | 68135681 | 68135726 | 68135756 | 68135823 |
| Hap1 | A | G | G | C | G | G | A | C | G |
| Hap2 | A | G | A | C | G | A | G | C | G |
| Hap3 | A | T | A | C | G | A | G | C | G |
| Hap4 | A | G | A | C | G | G | G | T | G |
| Hap5 | G | G | G | T | A | G | A | C | T |

Table S6 All predicted genes in the LD block from 10636869 to 10852447 on Chr03.

| Chr | Gene ID | Start | Stop | Length | Description |
| --- | --- | --- | --- | --- | --- |
| Chr03 | Sobic.003G117850 | 10638817 | 10639597 | 781 | Chloroplast Ycf2;ATPase, AAA type, core |
| Chr03 | Sobic.003G117900 | 10663773 | 10668027 | 4255 | Protein kinase superfamily protein |
| Chr03 | Sobic.003G118000 | 10669660 | 10670849 | 1190 | NA |
| Chr03 | Sobic.003G118100 | 10671722 | 10679149 | 7428 | Protein kinase family protein with ARM repeat domain |
| Chr03 | Sobic.003G118200 | 10680982 | 10688310 | 7329 | phosphoadenosine phosphosulfate (PAPS) reductase family protein |
| Chr03 | Sobic.003G118266 | 10693336 | 10696116 | 2781 | Chalcone and stilbene synthase family protein |
| Chr03 | Sobic.003G118332 | 10707828 | 10712129 | 4302 | nodulin MtN21 /EamA-like transporter family protein |
| Chr03 | Sobic.003G118400 | 10717231 | 10721566 | 4336 | NA |
| Chr03 | Sobic.003G118500 | 10720812 | 10724035 | 3224 | F-box/RNI-like superfamily protein |
| Chr03 | Sobic.003G118600 | 10728053 | 10734964 | 6912 | F-box/RNI-like superfamily protein |
| Chr03 | Sobic.003G118650 | 10739016 | 10746036 | 7021 | Galactosyltransferase family protein |
| Chr03 | Sobic.003G118700 | 10747336 | 10748712 | 1377 | NA |
| Chr03 | Sobic.003G118750 | 10746027 | 10748963 | 2937 | NA |
| Chr03 | Sobic.003G118800 | 10754658 | 10757309 | 2652 | NA |
| Chr03 | Sobic.003G118900 | 10759326 | 10763174 | 3849 | LRR and NB-ARC domains-containing disease resistance protein |
| Chr03 | Sobic.003G119000 | 10764024 | 10773174 | 9151 | 5\'-3\' exoribonuclease 3 |
| Chr03 | Sobic.003G119100 | 10777291 | 10779414 | 2124 | Pentatricopeptide repeat (PPR) superfamily protein |
| Chr03 | Sobic.003G119200 | 10779450 | 10785977 | 6528 | P-loop containing nucleoside triphosphate hydrolases superfamily |
| Chr03 | Sobic.003G119266 | 10785393 | 10787707 | 2315 | NA |
| Chr03 | Sobic.003G119332 | 10791911 | 10793525 | 1615 | NA |
| Chr03 | Sobic.003G119400 | 10802232 | 10806033 | 3802 | purin-rich alpha 1 |
| Chr03 | Sobic.003G119500 | 10815064 | 10816875 | 1812 | NA |
| Chr03 | Sobic.003G119600 | 10833175 | 10838076 | 4902 | RING/U-box superfamily protein |

Table S7 All predicted genes in the LD block from 68016493 to 68153310 on Chr04.

| Chr | Gene ID | Start | Stop | Length | Description |
| --- | --- | --- | --- | --- | --- |
| Chr04 | Sobic.004G351600 | 68019739 | 68025379 | 5641 | NA |
| Chr04 | Sobic.004G351700 | 68033516 | 68035880 | 2365 | C2H2-type zinc finger family protein |
| Chr04 | Sobic.004G351800 | 68053030 | 68055363 | 2334 | Tetratricopeptide repeat (TPR)-like superfamily protein |
| Chr04 | Sobic.004G351866 | 68056486 | 68059151 | 2666 | NA |
| Chr04 | Sobic.004G351932 | 68059519 | 68061151 | 1633 | F-box and associated interaction domains-containing protein |
| Chr04 | Sobic.004G352000 | 68079980 | 68082101 | 2122 | cytochrome P450, family 715, subfamily A, polypeptide 1 |
| Chr04 | Sobic.004G352100 | 68085623 | 68090287 | 4665 | AT hook motif DNA-binding family protein |
| Chr04 | Sobic.004G352200 | 68094407 | 68096848 | 2442 | NA |
| Chr04 | Sobic.004G352301 | 68097411 | 68098526 | 1116 | Remorin family protein |
| Chr04 | Sobic.004G352400 | 68098558 | 68099971 | 1414 | NA |
| Chr04 | Sobic.004G352500 | 68101898 | 68106149 | 4252 | vacuolar ATPase subunit F family protein |
| Chr04 | Sobic.004G352600 | 68106336 | 68110980 | 4645 | DEA(D/H)-box RNA helicase family protein |
| Chr04 | Sobic.004G352700 | 68110772 | 68113362 | 2591 | NAD(P)-binding Rossmann-fold superfamily protein |
| Chr04 | Sobic.004G352750 | 68114420 | 68116102 | 1683 | NA |
| Chr04 | Sobic.004G352800 | 68116426 | 68118036 | 1611 | NA |
| Chr04 | Sobic.004G352900 | 68118781 | 68120899 | 2119 | NA |
| Chr04 | Sobic.004G353000 | 68121245 | 68127531 | 6287 | general control non-repressible 3 |
| Chr04 | Sobic.004G353100 | 68129916 | 68134915 | 5000 | Glucose-6-phosphate translocator |
| Chr04 | Sobic.004G353200 | 68135946 | 68140167 | 4222 | 26S proteasome regulatory subunit, putative (RPN5) |
| Chr04 | Sobic.004G353300 | 68140107 | 68146286 | 6180 | O-fucosyltransferase family protein |
| Chr04 | Sobic.004G353400 | 68146887 | 68152858 | 5972 | Leucine-rich repeat (LRR) family protein |
